# A Mobile Phone App Intervention Targeting Fruit and Vegetable Consumption: The Efficacy of Textual and Auditory Tailored Health Information Tested in a Randomized Controlled Trial

**DOI:** 10.2196/jmir.5056

**Published:** 2016-06-10

**Authors:** Sarah Pietertje Elbert, Arie Dijkstra, Anke Oenema

**Affiliations:** ^1^ Department of Social Psychology University of Groningen Groningen Netherlands; ^2^ CAPHRI School for Public Health and Primary Care Department of Health Promotion Maastricht University Maastricht Netherlands

**Keywords:** mobile phone app, health behavior, fruit and vegetable intake, persuasive communication, communication modality, audio, intervention study

## Abstract

**Background:**

Mobile phone apps are increasingly used to deliver health interventions, which provide the opportunity to present health information via different communication modes. However, scientific evidence regarding the effects of such health apps is scarce.

**Objective:**

In a randomized controlled trial, we tested the efficacy of a 6-month intervention delivered via a mobile phone app that communicated either textual or auditory tailored health information aimed at stimulating fruit and vegetable intake. A control condition in which no health information was given was added. Perceived own health and health literacy were included as moderators to assess for which groups the interventions could possibly lead to health behavior change.

**Methods:**

After downloading the mobile phone app, respondents were exposed monthly to either text-based or audio-based tailored health information and feedback over a period of 6 months via the mobile phone app. In addition, respondents in the control condition only completed the baseline and posttest measures. Within a community sample (online recruitment), self-reported fruit and vegetable intake at 6-month follow-up was our primary outcome measure.

**Results:**

In total, 146 respondents (ranging from 40 to 58 per condition) completed the study (attrition rate 55%). A significant main effect of condition was found on fruit intake (*P*=.049, partial η^2^=0.04). A higher fruit intake was found after exposure to the auditory information, especially in recipients with a poor perceived own health (*P*=.003, partial η^2^=0.08). In addition, health literacy moderated the effect of condition on vegetable intake 6 months later (*P*<.001, partial η^2^=.11). A higher vegetable intake was found for recipients with high health literacy after exposure to the textual or auditory intervention compared to the control condition (contrasts *P*=.07 and *P*=.004, respectively). In the case of relatively low health literacy, vegetable intake was the highest in the control condition (contrasts text control: *P*=.03; audio control: *P*=.04).

**Conclusions:**

This study provides evidence-based insight into the effects of a mobile health app. The app seems to have the potential to change fruit and vegetable intake up to 6 months later, at least for specific groups. We found different effects for fruit and vegetable intake, respectively, suggesting that different underlying psychological mechanisms are associated with these specific behaviors. Based on our results, it seems worthwhile to investigate additional ways to increase fruit and vegetable intake in recipients with low health literacy.

**ClinicalTrial:**

International Standard Randomized Controlled Trial Number (ISRCTN): 23466915; http://www.isrctn.com/ISRCTN23466915 (Archived by WebCite at http://www.webcitation.org/6hTtfSvaz)

## Introduction

The number of mobile health apps is increasing rapidly. In 2013, more than 30,000 health apps were available [1], whereas more than 150,000 health-related apps were found only two years later [2]. In the broad context of mHealth, apps have been developed and used in health interventions, focusing on a wide array of behaviors and having different functions, such as the prevention and management of chronic and mental illnesses [3], and education about smoking, physical activity, or nutrition in an effort to stimulate behavior change in these domains [4,5]. However, scientific and evidence-based research with regard to the efficacy of these mobile health apps is lacking [3,6,7]. A recent review [6] shows that most published work on mobile apps used in health behavior change interventions are pilot studies [8] or describe the app in terms of content or acceptability [5,9]. Moreover, mobile phone apps are often offered as an additional tool to stimulate health behavior change next to an eHealth intervention, face-to-face counseling, or a virtual coach. To the best of our knowledge, only a few randomized controlled trials exist that focus on the effects of standalone mobile health apps [10,11], with only minimal follow-up periods ranging from 6 to 8 weeks. This means there is limited knowledge on the effectiveness of mobile phone apps in the process of health behavior change. In this study, the effects of auditory and textually tailored health information provided via a mobile phone app are tested in a randomized controlled trial.

Using mobile phone apps to deliver an intervention can have a variety of advantages. In addition to the increased availability and accessibility of mobile phones and the potential of reaching many people, it provides the opportunity to use interactive technological possibilities for persuasion that may support behavior change [12]. In particular, it enables the use of different communication modes (eg, text, video, and audio) and the use of computer tailoring to convey health information [13]. Mobile phones are, in general, already partly used for their MP3 function and mobile phone apps can be easily used to include and deliver auditory information, such as integrated within a health intervention. In addition, there is some evidence that at least audiovisual tailored messages can have advantages compared to text-based tailored messages [14,15] and at least one study suggests that audio-based information may be of added value in the stimulation of fruit and vegetable consumption [16]. Furthermore, tailoring can have beneficial effects in health interventions over providing nontailored information, for example, by increasing the relevance of the information [17-20]. The goal of this study is to investigate the efficacy of a mobile phone intervention that delivers tailored persuasive information as communicated via two different communication modes: text versus audio.

The mode of communication via which the persuasive health information is delivered might affect how the information is processed. For instance, compared to textually tailored information, interactive tailored information (either video- or audio-delivered) has been found to lead to greater attention [21,22] and is perceived as being more salient [23] and engaging [22]. In addition, in processing video- and audio-delivered communication, source considerations and peripheral cues or heuristics may play a more important role [24]. Furthermore, one study showed no significant differences between auditory and textual feedback on the recall of health-related information [25]. Other studies found mixed results between audiovisual and textual feedback [21,25]; audio only or audiovisual information was not always more effective than textual feedback. Thus, concerning the communication of health-related information, no explicit conclusion can be formulated with regard to the efficacy of a specific communication mode.

This intervention will apply and compare auditory and textual persuasive communication aimed at stimulating fruit and vegetable intake. A sufficient daily intake of fruit and vegetables contributes to the prevention of cardiovascular diseases and certain types of cancer [26]. However, more than 70% of the Dutch adult population does not meet the recommended minimum intake of fruit and this percentage is even higher for vegetable consumption [27]. These recommendations refer to a daily consumption of two pieces of fruit and 200 grams of vegetables for an adult population [28]. In addition, the average intake levels of fruit and vegetables seems to be decreasing over the years [27]. Moreover, similar intake patterns and trends are identified all over the world [29]. Thus, the stimulation of fruit and vegetable consumption remains a highly important health promotion topic.

To determine which communication mode can be used to deliver the tailored health information most effectively, it is important to test this in a randomized controlled trial. In this study, two research questions will be central. First, we aim to answer the question whether a tailored health intervention delivered via a mobile phone app is able to change fruit and vegetable intake in the advocated direction. Second, this study provides an exploratory test of the possible difference in efficacy between the more classic textual mode of communication (reading) and the auditory mode of communication (listening).

With regard to the first research question, it is expected that a tailored health intervention will be more effective compared to a control condition in which no health information is given. However, this difference may not be displayed in everyone, but only in a specific group of people. It is hypothesized that this will be especially the case in people who perceive a need to change their fruit and vegetable intake. It is reasoned that people who perceive their own health as relatively good have a lower need to change, whereas this need is higher for people who perceive their own health as relatively poor. The intervention might fit within the need for this latter group and, therefore, might be more beneficial for people who perceive their own health as relatively poor. Within the unimodel of persuasion [30], this could be described as a match between the persuasive information and a premise held by a person (eg, “I might need this information because my health is not that good”), whereas this match might be lacking for people who perceive their own health as good in advance. Therefore, we will test the hypothesis that the intervention (either textual or auditory) will be more effective, especially for people who perceive their own health as relatively poor.

With regard to the second (exploratory) research question, it will be investigated whether the efficacy of the auditory intervention differs from the textual intervention. Again, this might not be the case for everyone. A relevant individual difference in this context is health literacy, defined as “the degree to which individuals can obtain, process, and understand the basic health information and services they need to make appropriate health decisions” [31]. Furthermore, health literacy is found to be related to level of education, cognitive and social skills, language and cultural barriers, and motivation [31,32], and low health literacy is associated with poorer health outcomes as well [33,34]. It seems worthwhile to consider the communication modality in combination with the construct of health literacy [34]. For instance, it is recommended to explore the use of auditory information because this might be especially beneficial for people with low health literacy [32]. Therefore, it is expected that people with low health literacy may benefit from health information communicated via the auditory mode, whereas no specific differences are expected for people with high health literacy.

In summary, we aim to test the efficacy of two different fruit and vegetable promotion interventions delivered via a mobile phone app that communicates persuasive health information via an auditory or textual mode. The efficacy of the auditory and textual intervention will be compared with a control condition in which no intervention is present, and the textual and auditory interventions will be compared to each other. The content of the intervention is tailored to relevant characteristics of the individual: Feedback on the participants’ perceived fruit and vegetable consumption and personalized recommendations regarding the individual barriers to eating sufficient fruit and vegetables are included. Other evidence-based behavior change strategies [35] applied in this intervention to assist behavior change are listed in Table 1. The dependent variables are self-reported fruit intake and self-reported vegetable intake at 6-month follow-up as assessed with a detailed and validated frequency questionnaire [36].

**Table 1:** Overview of behavior change techniques applied in the intervention.

Behavior change technique		Example
**Tailored working mechanisms**		
	Provide feedback on performance	“You indicate that you eat sufficient fruit and vegetables, that is very positive” (baseline message)
	Adaptation of the content	Provide information on how to overcome personal barriers; the inclusion of information about weight management based on dieting status (baseline message)
	Preference tailoring	Use of preferred conversational form throughout the intervention
	Testimonial matching	Exposure to a testimonial of the same gender as the respondent (follow-up moments)
**General working mechanisms**		
	Provide information about behavior-health link	Provide general health risk information: “Eating sufficient fruit and vegetables contributes to good health” (baseline message)
	Provide information on consequences	Provide positive outcomes of performing the behavior: “The vitamins, minerals, and fibers in fruit and vegetables affect your health in several ways” (low blood pressure, improved physical stamina, and decreased risk for diseases; baseline message)
	Barrier identification	“Different aspects can play a role in eating insufficient fruit and/or vegetables. Of the following reasons, can you list maximally two aspects that apply to you?” (assessed at baseline)
	Use of follow-up prompts	Monthly follow-up moments are created to encourage respondents to revisit the app
	Provide opportunities for social comparison	The use of testimonials (follow-up moments)
	Prompt specific goal setting	Respondents are encouraged to create own implementation intentions (general app content; menu button “action plan”)
	Repetition	Respondents can be exposed to the tailored message multiple times (general app content; menu button “my advice”)

## Methods

### Recruitment

Participants were recruited in October and November 2013 to join a mobile phone intervention study aimed at stimulating fruit and vegetable intake. Those interested were eligible for participation if they were 16 years or older, lived in the Netherlands, and owned an Android device (mobile phone or tablet, Android version 2.2 or later) with an installed version of Adobe Air (if necessary, they were automatically directed to Google Play to install it safely). In collaboration with the programmers (affiliated with the University of Groningen), we decided to focus solely on the Android operating system because market research showed that the majority of Dutch mobile phone users owned an Android device [37]. In addition, the recruitment invitation was specifically aimed at people who had not (yet) succeeded in consuming two pieces of fruit and 200 grams of vegetables on a daily basis. After the 2-month recruitment period, interested people who signed up could not participate anymore.

Participants were recruited via several advertising campaigns published on newspaper and (health) magazine websites, on the local university website, in the online newsletter of the Netherlands Nutrition Centre, and via social networking websites. In addition, a local newspaper focused on the topic of fruit and vegetable consumption and referred to our mobile phone app, the “Fruit and Vegetables hAPP” (the addition of the “h” to the word “app” in Dutch means “snack” or “bite”). All advertisements briefly mentioned the content and 6-month duration of the study and provided a link to the mobile phone app in Google Play where respondents could find more information and download it. Respondents were not informed about the existence of different research conditions. They had a chance of winning different prizes (two Android tablet computers, 10 books with vegetable recipes, and 20 €10 coupons) after completing the pretest and posttest measurements. The study was approved by the ethical committee of the faculty of Behavioral and Social Sciences for conducting human participant research at the University of Groningen (no: 13012-N; trial registration: ISRCTN23466915).

We estimated the number of respondents to be included. This intervention and the comparison between auditory and textual persuasion were novel; therefore, it was difficult to predict what could be expected. We aimed to find medium effects for the comparison between one of the interventions and the control condition for the intervention to be of practical relevance. This meant at least 64 respondents needed to be included in each condition at posttest (*P*=.05, power=.80) [38].

### Research Design and Procedure

**Figure 1:**
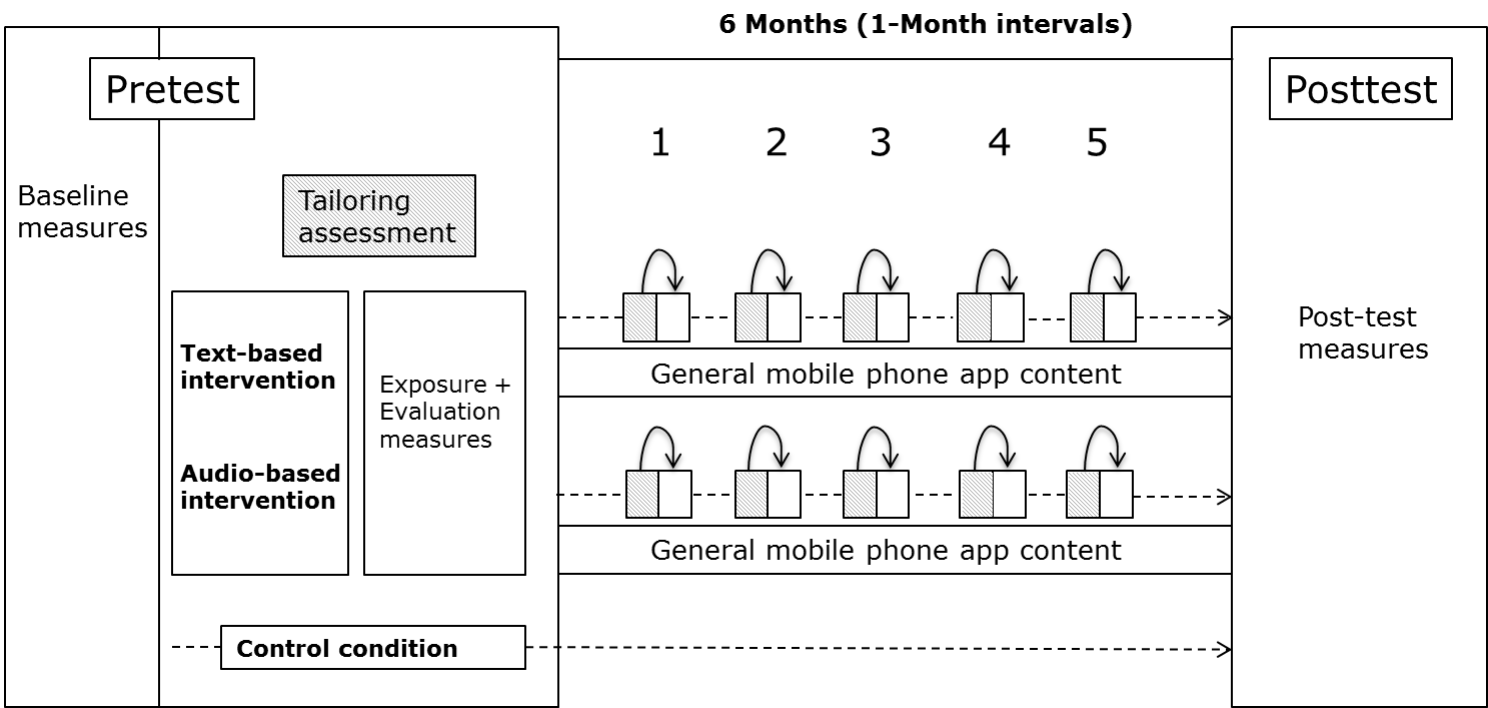
The design of the mobile phone health intervention to stimulate fruit and vegetable intake.

This study was a pretest-posttest randomized controlled trial with two experimental conditions (text-based tailored health information and audio-based tailored health information) and a control condition in which respondents completed only the baseline measurements and posttest measurements at the 6-month follow-up. Those interested could download the mobile phone app in Google Play and sign up for the research via the app itself. This was done by creating a personal account with an email address and password, which was necessary to combine the data of the different measurements. In addition, the sign-up procedure consisted of questions on gender, first name, and preferred conversation form (in Dutch, a formal and polite conversation form [*u, uw*] and a more informal conversation form [*jij, jou*] can be distinguished based on certain display rules). This information was used for tailoring purposes throughout the assessment and intervention. Next, respondents were presented with an informed consent form that stated the procedure, duration, and confidentiality of the research. In addition, it was mentioned that participation would be used for research purposes. After giving informed consent in the mobile phone app (with a checkbox), respondents were automatically assigned to one of the three conditions (sequentially in order of registration). All assessment questions (one question per screen) and the tailored health information were delivered via the mobile phone app. Figure 1 represents an overview of the design and the different elements of the study, which will be described subsequently.

Respondents in all conditions were asked to complete the pretest measures that consisted of baseline measures and questions for tailoring purposes. Respondents in the text-based and audio-based health information condition were then exposed to a tailored message (on the basis of decision rules) and additional evaluation measures. In total, this first contact took 20 minutes on average after which these respondents had access to the general mobile phone app content. Respondents in the control condition were only exposed to a message screen addressing the end of the baseline questionnaire. They were thanked for their participation and it was explicitly mentioned that they could expect another questionnaire 6 months later. They did not have access to the content of the mobile phone app.

Those respondents who did not complete the baseline questionnaire within 1 month were reminded by email to fill out the questionnaire. Further reminders were sent monthly; respondents who did not complete it during the research period were excluded from the study and informed about this by email.

In the months between the pretest and posttest assessments, respondents in the text-based and audio-based health information condition received monthly email invitations (with a maximum of four reminders during the month). They were asked to visit the mobile phone app to complete follow-up tailoring measures (identical for each month) and were exposed to newly added (either textual or auditory) tailored health information based on their input. Finally, at 6-month follow-up, all respondents were sent an email invitation to fill out the posttest measures in the mobile phone app (again with a maximum of four reminders during the month). Respondents who indicated they were interested in receiving more information were debriefed via email when the 6-month posttest had been completed. For ethical reasons, respondents could notify the researchers during the trial when they were not interested in participating anymore. After this notification, they did not receive monthly email invitations anymore, but only a final invitation to complete the posttest measures.

### Intervention

This intervention was developed in the framework of Intervention Mapping [39-41]. In addition, behavior change techniques [35] were applied and the intervention was based on several sociocognitive determinants known to predict fruit and vegetable intake [42]. The core determinants included outcome expectations of eating sufficient fruit and vegetables and self-efficacy with regard to being able to eat sufficient fruit and vegetables. More specifically, the intervention focused on increasing positive outcome expectations and restructuring negative outcome expectations with regard to eating sufficient fruit and vegetables, and increasing self-efficacy. Different methods were used to address these factors. For instance, to target self-efficacy, we identified the respondents’ experienced barriers to eating sufficient fruit and vegetables and provided relevant information to cope with these barriers [35].

**Figure 2:**
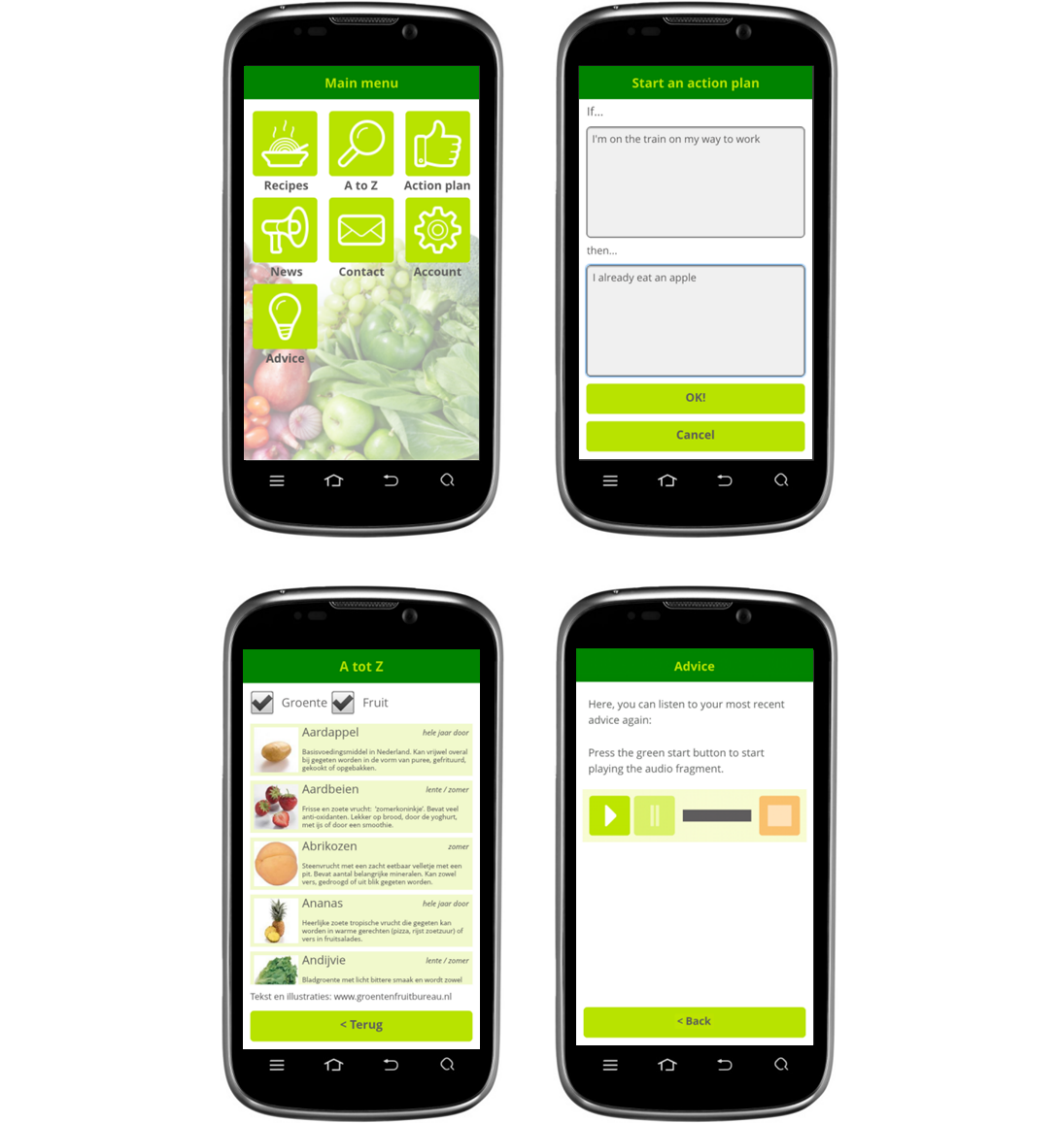
Screenshots of the app content: the main menu, starting an action plan, an alphabetical list of fruit and vegetables, and a tailored auditory advice. Three screenshots are translated from Dutch.

The intervention consisted of one main moment of exposure to the tailored information at baseline and five follow-up moments with exposure to smaller components of tailored information. After exposure to the main tailored message right after completing the pretest measurements, respondents had access to additional functions of the mobile phone app throughout the 6 months. These functions were presented as seven main menu buttons (a screenshot is presented in Figure 2), including a button that invited respondents to formulate a personal action plan while making use of “if...then” formulations (implementation intentions; Figure 2), a button with an alphabetical list of fruit and vegetables (Figure 2), and fruit and vegetable recipes. Four extra recipes were uploaded to the mobile phone app every month. In addition, one button included the most recent tailored message so it was possible to read or listen to it again (Figure 2).

During the five follow-up moments between the pretest and posttest assessments, respondents were exposed to a new, short tailored message each month that approached the topic of fruit and vegetable intake from distinct perspectives. Every month, the content was related to a general topic: the effect on well-being, the availability of fruit and vegetables, fruit and vegetables as a basic physical need, the lowered risk for chronic diseases, fruit and vegetable intake as a part of a healthy lifestyle, and objections people can have regarding fruit and vegetable intake, respectively. Additionally, a unique testimonial was included each month for sharing experiences on fruit and vegetable intake with the respondents. Testimonials are constructed stories in which successful personal experiences are shared to “directly or indirectly encourage the audience” to perform the behavior themselves [43]. For example, a physician elaborated on the relevance of a healthy diet and a nonexpert (without a specified occupation) expressed the experienced benefits of fruit and vegetables in the long term. To ensure that respondents were exposed to the information from different perspectives, the follow-up content was replaced in the mobile phone app at all five follow-up moments.

#### The Main Tailored Message

At baseline, a number of tailoring questions were included to partly determine the content of the feedback. Firstly, respondents could indicate with two questions whether their fruit and vegetable consumption was sufficient or not (perceived [subjective] consumption of fruit and vegetables, respectively), according to the recommendations. Participants could answer these questions with “Yes, I do meet this guideline;” “No, I probably do not always meet this guideline;” and “No, I do not meet this guideline.” The second category was added to prevent people from overreporting their fruit and vegetable intake; the latter two answering options both reflected “not meeting the guideline.”

Based on the answers given, respondents could select one or two individual barriers to eating sufficient fruit and/or vegetables from a predefined list. Respondents who indicated that they already met the guideline for eating sufficient fruit and vegetable consumption were asked to think of barriers in a future period in which they possibly would eat less fruit or/and vegetables. Examples of barriers included in the list were “I don”t like the taste of fruit” or “It takes a lot of time and effort to prepare vegetables” [44,45].

Another tailoring question concerned health value was assessed with one item (“How important is health to you?”). Participants could indicate whether they believed health was 1=not the most important to them (eg, “It is important to me, but not the most important aspect in life”) or 2=most important to them. In addition, they could indicate the perceived difference between themselves and their ideal and ought self, respectively (“In general, how large is the difference between who you actually are and who you prefer to be / who you should be?”). A scale was created by subtracting the score on the second item from the score on the first item; answering options ranged from 1=very small to 7=very large. The combination of health value and self-discrepancy determined whether the baseline message focused on the positive outcomes of sufficient fruit and vegetable consumption or on the negative outcomes of insufficient fruit and vegetable consumption [46]. Finally, it was assessed whether respondents were frequent dieters (answering options: 1=never, 2=sometimes, 3=regularly, 4=often) based on Lowe and Timko [47], and whether they had a partner relationship or not. These answers were used to decide whether or not to include information on the outcomes of sufficient/insufficient fruit and vegetable consumption related to weight management and appearance benefits, respectively.

Based on the answers on the previously mentioned tailoring questions, the baseline message consisted of a short general introduction providing information on the behavior-health link, feedback on their own fruit and vegetable consumption, and adapted information on the outcomes of sufficient/insufficient fruit and vegetable consumption with possibly information about appearance benefits and weight management. Then, feedback regarding one or two assessed individual barriers to perform the behavior with personal recommendations and formulation of relevant individual implementation intentions [48-50] were included. Throughout the mobile phone app, the preferred conversation form was used consistently. Transition sentences and closing sentences were created to ensure that the composed message was perceived as one fluent message.

#### The Follow-Up Tailored Messages

During all follow-up moments, respondents had to answer a maximum of four tailoring questions. First, the two questions on the current self-perceived fruit and vegetable intake were assessed again. The recommendations were included and respondents could indicate whether they met this guideline in the previous two weeks (answering options: yes/almost/no; the latter two answering options were considered as “not meeting the guideline”). In case of insufficient self-perceived fruit and/or vegetable consumption, we additionally asked whether the respondent had the intention to increase fruit and/or vegetable consumption in the following two weeks (answering options: no/a little/yes; the first two options reflected “no intention”).

Based on the given answers, respondents were then exposed to a short (textual or auditory) feedback message that addressed a certain theme at each follow-up moment. After this, the (textual or auditory) testimonial was included that matched the respondent’s own gender, except when it was one of the three testimonials that were only recorded with either a male or a female voice. This was decided in line with general expectations; for example, a dietician was only represented by a female voice. Respondents who already perceived their fruit and vegetable consumption as sufficient were not exposed to the thematic information, but only to a short encouraging message and testimonial.

#### Mode of Delivery

The text-based and audio-based interventions varied only in their mode of delivery. The content information was partly composed in collaboration with the Netherlands Nutrition Centre and the auditory elements were developed in collaboration with a professional recording studio. An experienced female actor was selected for recording the baseline and follow-up feedback messages. She had a gender-congruent voice (feminine and high-pitched) and neutral sound without specific cultural or disturbing elements. After recording and arrangement sessions, the tailored audio files (233 files for the pretest and 114 for the follow-up moments, ranging from a single sentence to a text of 200 words) were mastered in 96 kHz 24 bit and converted to mono MP3 format (64 kbps) to use in the mobile phone app. Because it was important that the audio files of different parts could be arranged into one fluent message (without experiencing obvious transitions between parts), it was ensured that all recordings had a similar “tone of voice.” Natural pauses between sentences lasted approximately 1 second; after every part, a 1-second pause was created as well to create as natural transition as possible. After a first evaluation round, the recording studio made some improvements; once the first author approved this, the audio files were uploaded in the intervention system by the programmers. Before listening to the baseline message, respondents in the audio-based information condition were presented with an instructive recording on volume regulation. They could adjust the mobile phone volume while listening to ascertain that it was sufficient and convenient. On the next screen, they could listen to the tailored health message. The complete message at baseline consisted on average of approximately 900 words (approximately 5 minutes for the auditory recording), roughly varying between 600 words (approximately 3 minutes) and 1200 words (approximately 6 minutes). In addition, the shorter tailored messages at follow-up consisted of 180 words on average and lasted 1:10 minutes on average for the auditory recording.

Contrary to the other auditory content, the testimonials within the follow-up moments were recorded with nonprofessional voices. The first author gave instructions and the testimonials were recorded in an office environment with a headphone microphone with the Praat software program [51] and arranged with the Audacity software program [52]. The recordings were send to the professional recording studio to make sure that the testimonials had the same quality and default volume as the remaining auditory content, again to ensure that it could be composed together. In total, 11 expert and nonexpert testimonials were created, among which four were recorded twice (ie, with a male and female voice). Three testimonials were only recorded for a man or a woman. On average, the testimonials consisted of 244 words and lasted 73 seconds. In total, an average follow-up moment lasted 2 to 3 minutes.

Multimedia Appendix 1 contains video and audio material of the mobile phone app content to provide insight into the registration procedure, baseline assessment, and exposure to the information at baseline and follow-up.

### Measurements

#### Baseline Measurement

The following sociodemographic variables were assessed: age, cultural background, and highest level of completed education. This latter item was dichotomized into low (primary education, lower general secondary education, intermediate vocational education) and high level of completed education (higher general secondary education, higher vocational education, university level). Then, health-related questions were asked. The participants’ perceived own health [53] was indicated on a 6-point scale ranging from (“my health is...”) 1=very good to 6=very bad. This item was recoded and high scores corresponded with good perceived health (mean 4.86, SD 0.72). Self-reported height and weight were assessed to calculate body mass index (BMI) and we assessed whether respondents had a chronic disease or dyslexia. In addition, two items assessed perceived difficulty of eating sufficient fruit and sufficient vegetables as a measure of self-efficacy: “How difficult is it for you to eat sufficient fruit (vegetables)?” Both items could be answered on 5-point scales (1=not difficult at all, 2=not difficult, 3=neutral, 4=difficult, 5=very difficult). A composite measurement was created (*r*=.37, *P*<.001; mean 2.47, SD 0.95).

Then, we assessed the self-reported fruit and vegetable consumption in the previous month with a detailed and validated food-frequency questionnaire [36]. Respondents were asked how often, on average, per week they ate or drank products from several fruit and vegetable categories during the previous month. The answer options ranged from 0=never or less than 1 day a week, 1=1 day a week, to 7=every day. Next, they were asked to indicate the amount of intake per category of fruit or vegetables in terms of pieces of fruit and servings of vegetables, with the answering options ranging from 0=none, 1=1 piece, to 5=5 or more pieces. The main categories were cooked vegetables, raw vegetables/salad, fruit and vegetable juice, tangerines, oranges/grapefruits/lemons, apples/pears, bananas, other fruit, and apple sauce. The category “fruit and vegetable juice” was excluded because we would be unable to distinguish between fruit and vegetables. The number of days per week and the vegetable portions were multiplied for the first two categories and added to create a composite index of mean weekly vegetable intake for the previous month. The mean number of days per week and the fruit portions were multiplied for the remaining six categories and added to create a composite index of weekly fruit intake for the previous month.

Finally, we assessed respondents’ health literacy with three statements that could be answered on 5-point scales ranging from 1=strongly disagree to 5=strongly agree. The three items were “I think it is easy to understand...information about health and lifestyle” / “...health information given by a physician, for example about a disease or treatment” / “...information about the effects of healthy nutrition” (α=.85, mean 4.23, SD 0.77).

Process evaluation questions were included immediately after respondents in one of the two experimental conditions were exposed to the tailored health information. These items were included to assess self-reported exposure (“Did you read/listen to the fragment?” answering options ranging from 1=yes, completely to 5=no, not at all) and potential distracting elements while reading or listening (“Was the reading or listening possibly disrupted, for example, by other people, hard sounds, music, or other distracting elements?” with answering options “yes” and “no”). In addition, the novelty and usefulness of the information were assessed with two statements (“The information was new to me / useful for me”) that could be answered on a 5-point scale ranging from 1=strongly disagree to 5=strongly agree. Finally, a general evaluation question was included (“How would you rate the intervention?”). This item could be answered on a 7-point scale ranging from 1=very negative to 7=very positive.

#### Posttest Measurement

At 6-month follow-up, fruit and vegetable intake was again assessed with the same questionnaire as at baseline [36]. Again, two composite measures for fruit and vegetable consumption, respectively, were created. In addition, it was assessed how often respondents searched for information about health and fruit and vegetables besides the information in the mobile phone app (mean 3.03, SD 1.03) and to what extent they spoke to others about the topic in the past 6 months (mean 2.71, SD 1.08). Both items could be answered on 5-point scales with answering options ranging from 1=never to 5=often. Finally, seven questions were added to evaluate the information and mobile phone app as a whole for a range of measures, such as personal applicability, novelty, credibility, the extent to which it is perceived as intense, usefulness, comprehensibility, and visual attractiveness. These questions could be answered on 5-point scales, with answering options ranging from 1=strongly disagree to 5=strongly agree. In addition, one item provided recipients with the opportunity to give qualitative feedback.

### Analyses

First, univariate analyses (ANOVA, chi-square) were conducted to analyze whether the respondents in the conditions differed on relevant pretest measures and to see whether respondents who dropped out after baseline (184/329, 55.9%) significantly differed from the respondents who completed both measurements. Second, ANCOVAs were conducted for fruit and vegetable intake separately, while controlling for self-reported fruit or vegetable intake at baseline, age, and highest completed education (because these two latter variables had a large variance within our community sample). After testing the main effects of condition, two moderators (perceived own health status and health literacy) were tested on fruit and vegetable intake to assess whether the effects of condition were similar in specific groups of respondents. The same covariates were included. To examine the meaning of the moderation effects, simple main analyses were conducted at two different levels (low/high) of the moderator. The complete dataset was then used to model respondents as scoring high or low by adding and subtracting 1 SD to the standardized means, respectively [54]. Post hoc contrasts were inspected to investigate the difference between one of the interventions and the control condition, and to explore differences between both interventions. In addition, in the intention-to-treat analyses, the posttest (T2) fruit and vegetable intake of respondents who did not complete the study were considered to be equal to their reported fruit and vegetable intake at pretest. With these data, we again conducted the analyses on fruit consumption and vegetable consumption. Finally, two categories of process measures were inspected referring to exposure to the intervention and a general evaluation of the main tailored message at baseline and the complete intervention. Age and education were now applied as covariates.

## Results

### Sample Characteristics

In total, 342 respondents registered for the study and started the pretest measurement and 96.5% completed it (330/342). Of these 330 respondents, 147 respondents (44.5%) completed the final questionnaire at 6-month follow-up as well. One respondent was excluded because he or she reported a fruit intolerance. The final sample for the analyses consisted of 146 respondents of whom 73.3% were females (n=107), 71.2% were highly educated (n=104), varied in age from 16 to 71 years (mean 41.4, SD 14.6), and with a mean BMI of 25.2 (SD 5.5). Recipients with a lower education reported a lower health literacy (mean 4.01, SD 0.77) compared to recipients with a higher education (mean 4.32, SD 0.76, *P*=.03). Thirty of 330 respondents (9.1%) were accidentally exposed to the same information during the first two follow-up moments (for technological reasons), instead of being exposed to different content information (referring to 20 respondents in the sample that actually completed the study, 13.7%).

The composite index of fruit and vegetable intake at pretest was treated as an indication of self-reported fruit and vegetable intake. The mean fruit intake was sufficient (14 portions considered sufficient; scale ranging from 0 to 56; mean 14.04, SD 10.63), whereas the mean vegetable intake was insufficient (28 portions considered sufficient; scale ranging from 6 to 70; mean 25.44, SD 11.37). If one of the two questions was answered with zero (“never or less than 1 day a week” or “no servings”), the total intake for that specific category was automatically set at zero as well (this was also when the pattern was not filled in consistently). This conservative approach means that the fruit and vegetable intake might be lower than in reality. Condition did not affect whether a question on fruit or vegetable intake was filled in consistently or not at baseline (*P*=.66) or posttest (*P*=.64).

**Figure 3:**
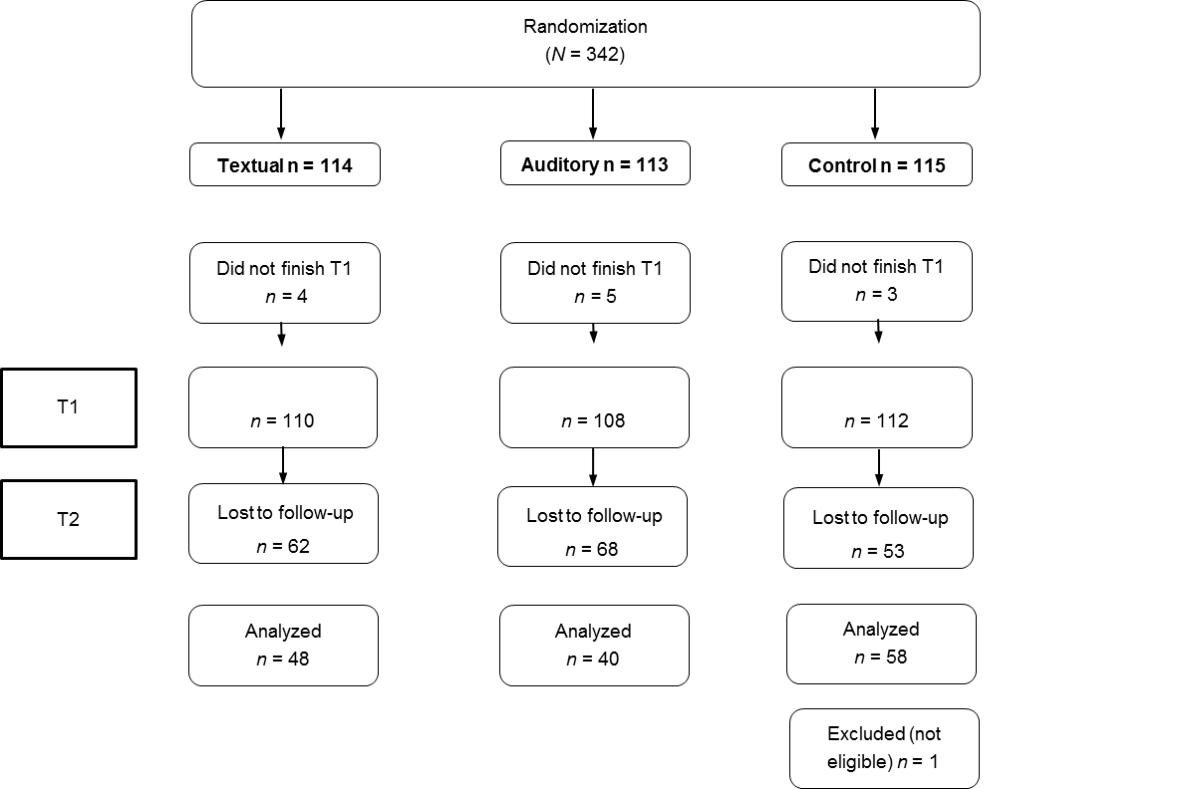
Flowchart of number of participants allocated per condition.

Based on the answers given, 19.2% (28/146) of the respondents were classified as consuming an insufficient amount of vegetables (but a sufficient amount of fruit), 16.4% (24/146) were classified as consuming an insufficient amount of fruit (but a sufficient amount of vegetables), 48.6% (71/146) were classified as consuming both insufficient amounts of fruit and vegetables, and 15.8% (23/146) were classified as consuming sufficient amounts of both fruit and vegetables. At posttest, the scores on the composite index of fruit and vegetable intake were somewhat higher (fruit: scale ranging from 1 to 56.5, mean 14.93, SD 9.27; vegetables: scale ranging from 6 to 70, mean 27.47, SD 11.81). The respondents were distributed over the conditions as follows: textual health information (n=48), auditory health information (n=40), control condition (n=58). Figure 3 represents a flowchart in which the dropout rates per condition are depicted.

### Randomization Check and Attrition Analyses

First, no significant differences between conditions were found regarding our set of 18 demographic and health-related baseline variables: gender (*P*=.53), age (*P*=.11), highest completed education (*P*=.35), cultural background (*P*=.75), dieting status (*P*=.09), relationship status (*P*=.41), the extent to which health is valued (*P*=.25), discrepancy between ought and ideal self (*P*=.54), having dyslexia (*P*=.94), perceived own health status (*P*=.78), having a chronic disease (*P*=.84), BMI (*P*=.30), self-efficacy (*P*=.72), self-reported fruit consumption (*P*=.79), self-reported vegetable consumption (*P*=.16), perceived (subjective) fruit and vegetable consumption (*P*=.86 and *P*=.37, respectively), and health literacy (*P*=.07).

In addition, the respondents who dropped out after baseline and the respondents who completed both measurements did not significantly differ on the pretest measures as mentioned in the previous paragraph (all *P*>.12). Furthermore, condition did not significantly affect whether respondents dropped out during the trial (*P*=.08). However, respondents who completed the study and who received either the auditory or textual feedback reported a significantly higher extent of being exposed to the information (*P*<.001) and they had a slightly more positive general impression of the pretest intervention content and measures compared to those who dropped out (*P*=.06). Finally, when exposed to the auditory feedback, respondents who dropped out reported being distracted while listening to the baseline intervention content more often compared to those who completed the whole study (*P*=.03). No significant differences were found on the extent to which the information was perceived as new or useful.

### Effects on Fruit and Vegetable Consumption

#### Main Effects

We assessed whether condition affected the self-reported intake of fruit and vegetables 6 months after baseline. With regard to fruit intake, a significant main effect was found (*F*_2,140_=3.08, *P*=.049, partial η^2^=0.04) with the following estimated means: text (mean 13.5, SE 1.0), audio (mean 17.1, SE 1.2), and control (mean 14.3, SE 0.9). The difference between text and audio was significant (*P*=.02), but it did not reach statistical significance between audio and control (*P*=.06). The difference between text and control was not significant (*P*=.53). No significant main effect was found on vegetable intake (*F*_2,140_=0.01, *P*=.99, partial η^2^=0.00).

For fruit consumption, the raw means at pretest and posttest were inspected to gain more insight into the actual differences per condition. The means remained quite similar in the textual feedback condition (pretest vs posttest: mean 14.8, SD 11.1 vs mean 14.2, SD 6.9) and in the control condition (pretest vs posttest: mean 13.4, SD 10.4 vs mean 13.8, SD 9.4). Thus, only small differences were observed here (mean –0.6 pieces and 0.4 pieces per week, respectively). In the auditory feedback condition, the fruit intake was most strongly increased (3.3 pieces; pretest vs posttest: mean 14.2, SD 10.6 vs mean 17.5, SD 11.1).

#### Moderation Effects

**Figure 4:**
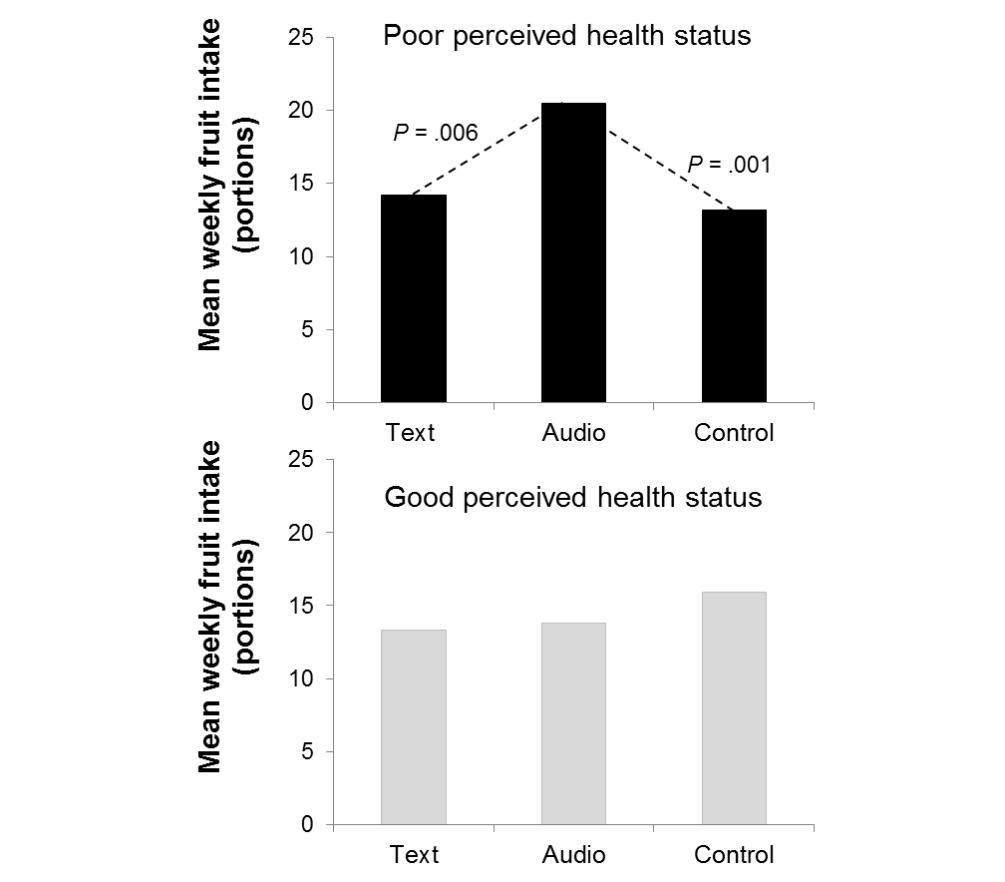
The interaction between condition and perceived own health status on fruit consumption at 6-month follow-up controlled for age, highest completed education, and self-reported fruit intake at baseline.

A significant interaction was found between condition and perceived own health status on fruit intake (*F*_2,137_=4.24, *P*=.02, partial η^2^=0.06), but not on vegetable intake (*F*_2,137_=0.15, *P*=.86, partial η^2^=0.00). Figure 4 displays the mean fruit consumption for respondents with poor and good perceived own health.

In case of poor perceived own health status, condition did significantly affect fruit consumption at 6-month follow-up (*F*_2,137_=6.05, *P*=.003, partial η^2^=0.08). The mean scores were as follows: text (mean 14.2), audio (mean 20.5), and control (mean 13.2). Post hoc contrasts showed that the intake of fruit was significantly higher after listening to the information compared to the other two conditions (text: *P=*.006; control: *P*=.001). In case of good perceived own health status, condition had no significant effect (*F*_2,137_=1.15, *P*=.32, partial η^2^=0.02). The mean scores were as follows: text (mean 13.3), audio (mean 13.8), and control (mean 15.9). No significant contrasts were found.

**Figure 5:**
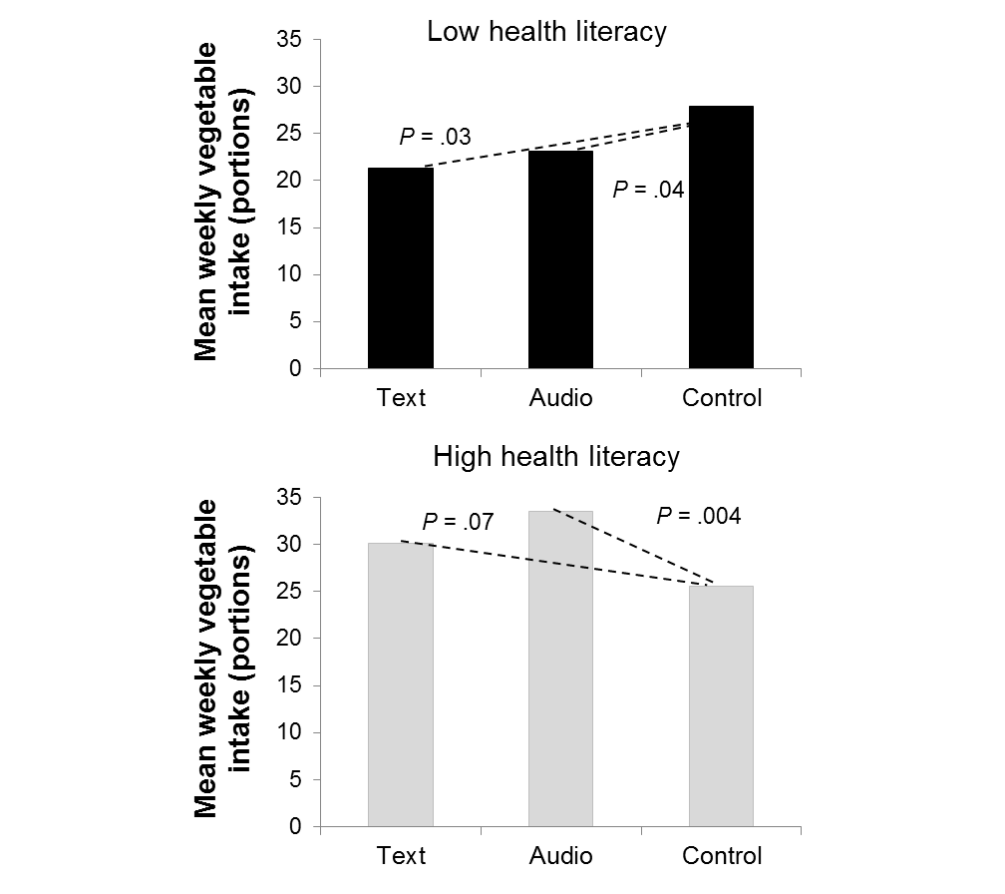
The interaction between condition and health literacy on vegetable consumption at 6-month follow-up controlled for age, highest completed education, and self-reported vegetable intake at baseline.

Second, health literacy as assessed at pretest was tested as a moderator. No significant interaction with condition was found on fruit intake (*F*_2,137_=0.25, *P*=.78, partial η^2^=0.00). However, we found a significant interaction on vegetable intake (*F*_2,137_=8.42, *P*<.001, partial η^2^=0.11). Figure 5 displays the mean vegetable intake in the conditions for people with relatively low and high health literacy.

In case of low health literacy, condition significantly affected vegetable consumption at 6-month follow-up (*F*_2,137_=3.62, *P*=.03, partial η^2^=0.05). The mean scores were as follows: text (mean 21.3), audio (mean 23.1), and control (mean 27.9). Post hoc contrasts showed that the intake of vegetables in this group was significantly higher in the control condition compared to the two interventions (text: *P*=.03; audio: *P*=.04). In case of high health literacy, condition significantly affected vegetable consumption at 6-month follow-up as well (*F*_2,137_=4.53, *P*=.01, partial η^2^=0.06). The mean scores were as follows: text (mean 30.1), audio (mean 33.5), and control (mean 25.6). In addition to the higher scores compared to respondents with low health literacy, post hoc contrasts showed that the intake of vegetables in this group was lower in the control condition compared to the textual intervention (*P*=.07) and the auditory intervention (*P*=.004).

The main effect of condition was not significant on a composite measure of fruit and vegetable consumption (*P*=.34) and neither was the interaction with perceived own health status (*P*=.16). However, the interaction with health literacy was (*F*_2,137_=4.39, *P*=.01, partial η^2^=0.06). The main effect of condition then became nonsignificant in respondents with low health literacy and only two of the four contrasts remained significant, showing that the control condition was most effective for low-literate respondents (compared to reading) and listening to the auditory information was most effective in high-literate respondents (compared to control).

We further analyzed the effects in selections of respondents for whom the health information could be especially relevant. With regard to the effects on fruit intake, respondents were selected whose self-reported fruit intake was found to be insufficient at pretest (and the vegetable intake was either insufficient or sufficient, n=95). The main effect on fruit consumption was not significant anymore (*F*_2,89_=1.85, *P*=.16, partial η^2^=0.04). The estimated means were lower showing a similar pattern: text (mean 11.2, SE 1.12), audio (mean 13.7, SE 1.25), and control (mean 10.9, SE 1.00). The contrasts were not significant anymore (all *P*>.07), but the pattern of findings remained similar. Again, this pattern was especially found for recipients with a poor perceived own health (moderation effect: *F*_2,86_=2.46, *P*=.09, partial η^2^=0.05; main effect: *F*_2,86_=3.95, *P*=.02, partial η^2^=0.08), showing significant differences between control (mean 8.9) and audio (mean 16.3; *P*=.006), and text (mean 10.5) and audio (*P*=.04) for this group. No significant differences between the conditions were found for recipients who perceived the own health as relatively good. As for the whole sample, health literacy did not moderate the effect on fruit intake.

In addition, the effects on vegetable intake were analyzed in a selection of respondents who indicated eating insufficient vegetables at pretest (and either insufficient or sufficient fruit intake, n=99). As in the whole sample, no significant main effect or moderation of perceived own health was found; however, the moderation of health literacy was not significant anymore (*F*_2,90_=0.07, *P*=.93, partial η^2^=0.00).

### Intention-to-Treat Analyses

At T2, 183 of 330 (55.5%) respondents had dropped out despite reminders to fill in the follow-up measurement. In the intention-to-treat analysis (while assuming that the fruit and vegetable intake was equal to the assessed fruit and vegetable intake at pretest for the respondents who dropped out), we again conducted the analyses on fruit consumption and vegetable consumption. The main effect on fruit consumption remained significant (*F*_2,323_=3.18, *P*=.04, partial η^2^=0.02) with the following means: text (mean 13.5, SE 0.52), audio (mean 15.3, SE 0.52), and control (mean 14.3, SE 0.51). Now, the interaction between condition and perceived health status on fruit consumption was not significant anymore (*P*=.33). However, as expected, for respondents with a poor perceived health status, the effect of condition remained significant (*P*=.04, partial η^2^=0.02) with a similar pattern of means and significant contrasts (audio vs text: *P*=.02; audio vs control: *P*=.04) compared to the original analyses. The interaction between condition and health literacy on vegetable consumption remained significant as well (*F*_2,320_=5.52, *P*=.004, partial η^2^=0.03). For both respondents with low and high health literacy, the effect of condition became marginally significant (all *P*=.07) with only the contrasts between audio-based health information and the control condition being significant (all *P*=.02). Overall, small(er) effect sizes were found.

### Process Analyses

Effects on two categories of process variables were inspected, referring to the exposure to the intervention and a general evaluation of the main tailored message at baseline and the intervention in general. First, with regard to exposure, participants logged in a mean 7.6 times (SD 4.5) and, as expected, this was significantly more in one of the experimental conditions (*F*_2,143_=42.11, *P*<.001, partial η^2^=0.37, text: mean 10.1, SD 3.7; audio: mean 9.4, SD 4.2) compared to the control condition (mean 4.2, SD 3.0; contrasts text control and audio control; both *P*<.001,). No significant difference was found between the two interventions (*P*=.35).

On average, respondents completed 4.1 follow-up moments (SD 1.36). There was a difference between the two interventions: after reading, recipients completed slightly more follow-up moments (mean 4.35, SD 1.19) compared to the recipients who listened to the information (mean 3.83, SD 1.50; *F*_1,86_=3.40, *P*=.07, partial η^2^=0.04). In addition, 55 of 88 (63%) respondents in one of the experimental conditions completed all five follow-up moments and at least two follow-up moments were completed by 84 of 88 (95%) of the respondents. Those respondents who completed four or five follow-up moments were selected (text: n=39; audio: n=25) and compared to the control group (n=58) with regard to the set of 18 baseline measures. The groups did not differ significantly from one another regarding these variables (all *P*>.06).

At baseline, respondents who were in one of the intervention conditions were asked to indicate the extent to which they were exposed to the main tailored message and the extent to which they experienced potential distracting elements. Slightly more respondents reported being only partly exposed after listening (*F*_1,214_=2.94, *P*=.09) and fewer respondents identified potential distracting elements after listening to the main tailored message compared to those who read the information (χ^2^_1_=2.7, *P*=.10).

Second, differences between conditions were found on perceived usefulness and the general evaluation of the main tailored message (*F*_1,214_=12.27, *P*=.001, partial η^2^=0.05 and *F*_1,214_=12.10, *P*=.001, partial η^2^=0.05, respectively): Respondents who *read* the baseline information experienced it as significantly more useful (mean 4.21, SE 0.08) and positive (mean 5.77, SE 0.09) compared to the respondents who *listened* to the baseline information (usefulness: mean 3.81, SE 0.08, general evaluation: mean 5.33, SE 0.09). No significant differences were found on the perceived novelty of the information (*P*=.29).

With regard to the evaluation of the mobile phone app and intervention content at the 6-month follow-up, respondents who were exposed to the audio-based content reported to have looked more often for additional information about health and fruit and vegetables mostly via Internet websites (*F*_2,141_=3.00, *P*=.05, partial η^2^=0.04) compared to respondents in the control condition (contrast *P*=.02). In addition, after exposure to the audio-based intervention respondents appeared to have talked more about the topic with other people (*F*_2,141_=2.49, *P*=.09, partial η^2^=0.03) compared to the control condition (contrast *P*=.03). When comparing both interventions, the feedback and the mobile phone app were experienced equally in terms of personal applicability, novelty, credibility, intensity, usefulness, comprehensibility, and visual attractiveness.

## Discussion

This study addressed the efficacy of two tailored mobile phone interventions in a sample of people who were invited to participate especially when they perceived their own fruit and vegetable intake as insufficient. The efficacy of the interventions was compared to a control condition in which no tailored health information was provided, and an exploratory comparison was made between the text-based and audio-based tailored intervention. Besides testing this main effect, two relevant moderators—perceived own health status and health literacy—were included in this research.

### Principal Results

It seems that the results for fruit consumption and vegetable consumption are different. The results on fruit consumption were supported by the similar findings of the intention-to-treat analysis and, although the effects were less strong in the selection of respondents, the pattern of findings was still present in respondents with a more objectively assessed fruit consumption that was insufficient (according to guidelines). The significant main effect showed that the audio-based intervention was more effective than both the text-based intervention and the control condition. The auditory mode of communication, but not the textual mode of communication, led to increased fruit consumption with a mean increase of three pieces of fruit a week. We did not expect specific differences between the efficacy of textual and auditory health information, but it is in line with previous studies on the potential efficacy of auditory information [16]. The auditory information may have led to more attention [21,22] or it may have been perceived as more rich and personal to the recipient [23], which is possibly translated into behavior change.

With regard to vegetable consumption, there was no significant main effect of condition. Instead, an interaction between condition and health literacy was found, which was supported by the intention-to-treat analysis, but the pattern of findings was not found in the selection of respondents with a more objectively assessed vegetable consumption that was insufficient. Yet, there was no difference between the auditory and textual health information: both the text-based and audio-based intervention led to higher vegetable consumption in respondents with high health literacy, whereas this was not the case for respondents with relatively low health literacy (within this highly educated sample). For them, both interventions led to a significant decrease in self-reported vegetable consumption at the 6-month follow-up compared to the control condition. It seems that the current mobile phone app was not helpful for people with relatively low health literacy, at least not in improving a complex behavior such as vegetable intake.

We found that respondents who perceived their own health as relatively poor reported higher fruit consumption after being exposed to the auditory health information. It was initially expected that the health information in general would be more relevant for recipients with a poor perceived own health because they might perceive the necessity to change and are willing to make more investments [43,55]. In other words, there is a match between the persuasive health information and a characteristic of the recipient [30]. Thus, it seems that recipients with poor perceived own health did benefit most from the rich and personal auditory information. Although speculating, this might be related to an optimal level of threat of the auditory information that was necessary to engage in behavior change or the promise that the threat will be lowered once the recipient engages in the behavior. It is important to address these underlying processes in further research because the findings on the current process evaluation measures are unlikely to explain this pattern.

The moderator effect on vegetable intake shows that the intervention in general seemed to have worked especially in respondents with high health literacy. It can be that recipients with relatively low health literacy did not understand all content information or were not motivated to process the information [32,34] and therefore discarded the information in general. For recipients with high health literacy, it did not seem to matter how the information was communicated because they may have been open to the content information regardless of the mode of communication. It is important to unravel the different aspects of health literacy; for instance, education could have played a relevant role because recipients with a lower education level also reported lower health literacy in this study, but it is also possible that low health literacy is related to a defensive reaction to threatening health information.

Thus, we observed a main effect on fruit intake and, within a subsample of respondents, we could also find effects on vegetable intake. However, this finding on vegetable intake could not be replicated within a subsample of recipients who indicated consuming insufficient vegetables. This suggests that the findings on vegetable intake are less robust than on fruit intake. In addition, according to a conventional rule of thumb [56], small to medium effect sizes are found for fruit intake ranging between partial η^2^=0.04 and partial η^2^=0.08. It remains the question whether the absence of a main effect on vegetable intake was a matter of power because the recommended number of respondents per condition (n=64) was not reached. Yet, this effect might then have been too small to be relevant and the moderating effect of health literacy on vegetable intake showing contradictory results for recipients with low and high health literacy may indicate that it is indeed unlikely to find a main effect on vegetable intake.

The results suggest that not everybody benefitted equally from the intervention and that it can even adversely affect vegetable consumption. Thus, the tailored health information may have negative effects in subgroups of respondents when the information is communicated either via a textual or auditory channel. This increased our awareness on possible side effects of persuasive health communication. For future research, it seems worthwhile to investigate how individual characteristics can be assessed to optimize the practice of persuasive health communication and to increase knowledge on how “hard-to-reach” groups can benefit from it as well [32]. Possibilities may not only lie in providing persuasive tailored health information via another communication modality (ie, video tailoring), but also in the use of other interactive methods and elements (ie, the use of sensors, serious games, or avatars).

We did not expect specific differences for fruit and vegetable intake. It may be that these differences are found because fruit and vegetables are products with different qualities with regard to taste, preparation and culinary uses [57], and perceived ease of increasing consumption [55]. It may be that after a follow-up period of 6 months, the auditory intervention was only able to change fruit intake because this is a relatively less difficult behavior to change. In addition, interventions may differentially lead to increased fruit and vegetable consumption [58]. For instance, the context in which the products are consumed might play a role: vegetables are more likely to be consumed in a social context (at dinner, with the rest of the family), whereas fruit is more likely to be consumed individually. Therefore, environmental interventions may be more effective for increasing vegetable intake. It seems a rational choice to assess fruit and vegetable consumption separately in future studies.

The effects on the process measures showed that there were no differences between the text-based and audio-based interventions with regard to exposure. However, more follow-up moments were completed by recipients who read the information compared to recipients who listened to the information. In addition, after the first contact, the baseline textual information was evaluated as more useful and positive compared to the auditory information. However, after 6 months the textual and auditory interventions were not evaluated significantly different on relevant measures, such as novelty, credibility, comprehensibility, and usefulness. Yet, recipients who were exposed to the auditory information searched more for health-related information and they discussed the topic more often with other people compared to the control condition. Further research is needed to explain these findings and to investigate how characteristics of the textual and auditory information may possibly have contributed to these differences.

### Limitations

This research also has several limitations. First, a high percentage of respondents did not complete the entire study, which might have biased these findings. High attrition rates are common in Internet-based health behavior change interventions [59,60]. Although initially one could reason that an app might lead to lower attrition rates because people can participate in the study anywhere and at any time, it seems that for mobile phone apps it is still a challenge to keep respondents involved after their first visit. This might illustrate the quick and shallow relationship people have with the Internet and mobile phone apps in general (“app snacking”). Yet, it is found that respondents who dropped out from the research did not differ in relevant pretest measures compared to the respondents who completed the study.

Although it was not a significant result in this study, more respondents dropped out in one of the intervention conditions, which is a common finding in health intervention research [60]. Respondents in the intervention conditions were sent email reminders frequently and they were informed about the possibility to end their participation via email, whereas this was not the case for respondents in the control condition. These aspects of our research might additionally have contributed to differences in attrition between the conditions. A specific improvement may refer to sending reminders as mobile phone notifications versus email messages.

Secondly, we aimed to increase exposure by sending email prompts and providing regular updates of intervention content [59]. However, it is difficult to detect the actual exposure to the intervention content. People may report that they were fully exposed to the information, but still we do not know the quality of the exposure. In addition, we could not test intervention components separately, which means we do not know specifically why respondents showed certain improvements. Thus, we were not able to determine the unique contribution of each component of the current mobile phone intervention and to make statements about the elements that affected fruit and vegetable intake specifically.

Thirdly, the sample was a selective community sample, which could have biased the results. Respondents were not necessarily a representation of the whole community because they were mostly highly educated with a Dutch nationality and had to use an Android device to be included in the research. Furthermore, in our recruitment, people were invited who did not eat sufficient fruit and vegetables, which is obviously a selection of respondents who might be interested in the topic of health and changing their health behavior. In addition, people tend to generally overestimate their fruit and vegetable intake with self-report assessment measures, as used in this study [61].

### Conclusions

In our view, this app may be an effective channel to change fruit and vegetable intake, at least in certain groups of respondents. The development of the audio-based content was more costly and time-consuming compared to the text-based content, but it has shown to have beneficial effects on fruit consumption, or at least for some subgroups. The results showed us that it is important to be aware of the possible side effects of psychological health interventions and to take into account individual differences when exposing respondents to threatening health information and personal feedback on fruit and vegetable intake.

A next step may be to optimize the mobile phone app. It is worthwhile to investigate possibilities to expose the subgroups to either one of the current interventions that was shown to be efficient, depending on the specific behavior one would like to change. Furthermore, tracking and sensor technologies can be added to use the mobile phone app as an intervention channel to its fullest potential [62], which means that recipients can keep track of their daily fruit and vegetable intake and may receive reminders to buy fruit and vegetables when they are in the supermarket. In addition, it would be worthwhile to ensure a higher level of interactivity between the recipient and the mobile phone app as an interactive information system.

To the best of our knowledge, this is a first test of the effects of communication modalities in an evidence-based tailored mobile phone app to stimulate fruit and vegetable intake. It provided us with new insights on the efficacy and processes involved, and we hope to inspire the testing and development of evidence-based mobile phone apps in the field of health education and promotion.

## Appendix

Multimedia Appendix 1 A powerpoint presentation with additional video and audio material of the smartphone application content.

Multimedia Appendix 2 CONSORT EHEALTH Checklist V1.6.

## Abbreviations

BMI: body mass index
