# Supplementary material for: A Mobile Phone App Intervention Targeting Fruit and Vegetable Consumption: The Efficacy of Textual and Auditory Tailored Health Information Tested in a Randomized Controlled Trial
Source: J Med Internet Res. 2016 Jun 10;18(6):e147. doi: 10.2196/jmir.5056 (PMC4920964; doi:10.2196/jmir.5056)
Supplement: Multimedia Appendix 1 [file jmir_v18i6e147_app1.pptx]

## Slide 1
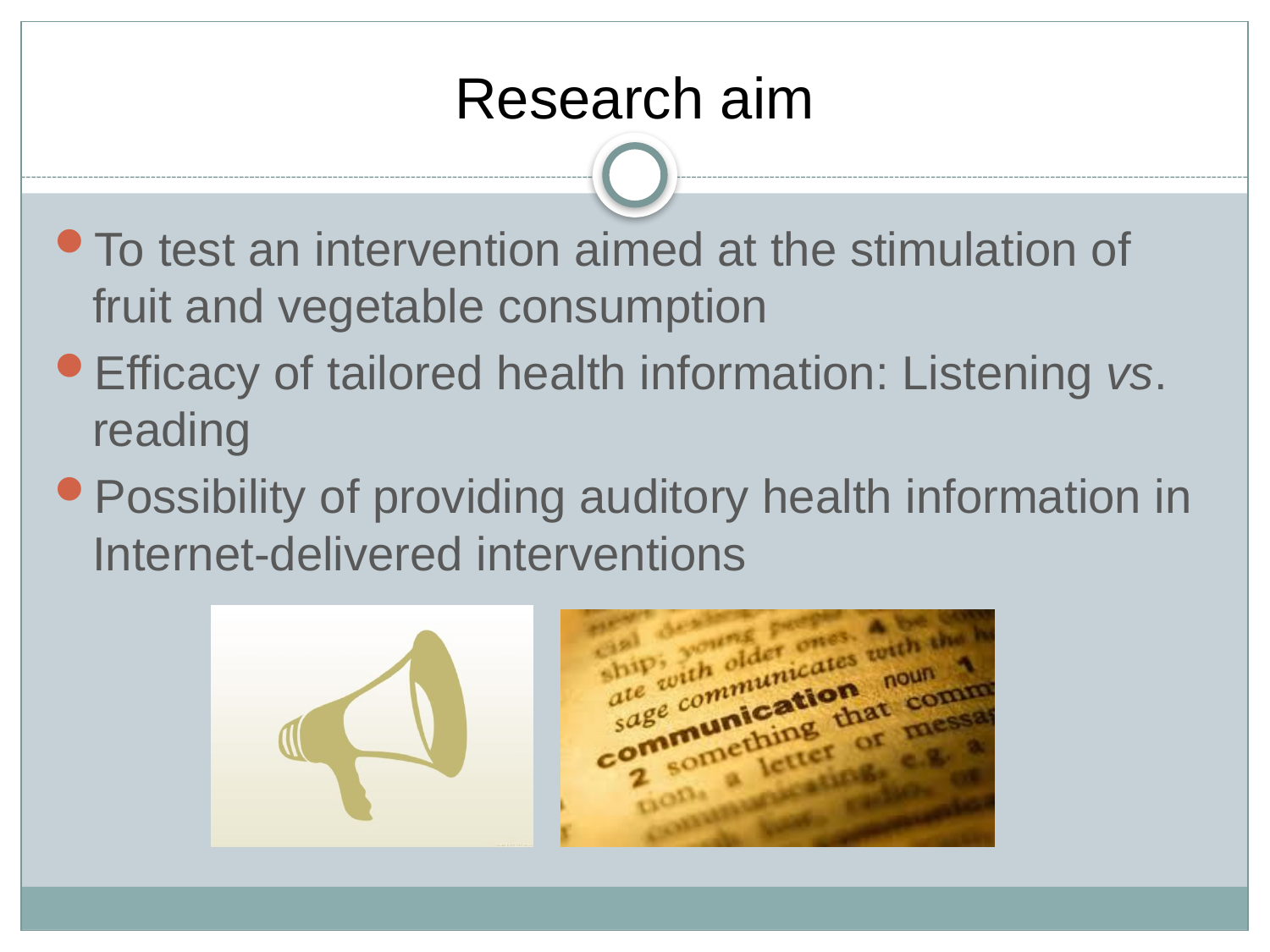

# Research aim
To test an intervention aimed at the stimulation of fruit and vegetable consumption
Efficacy of tailored health information: Listening vs. reading
Possibility of providing auditory health information in Internet-delivered interventions

## Slide 2
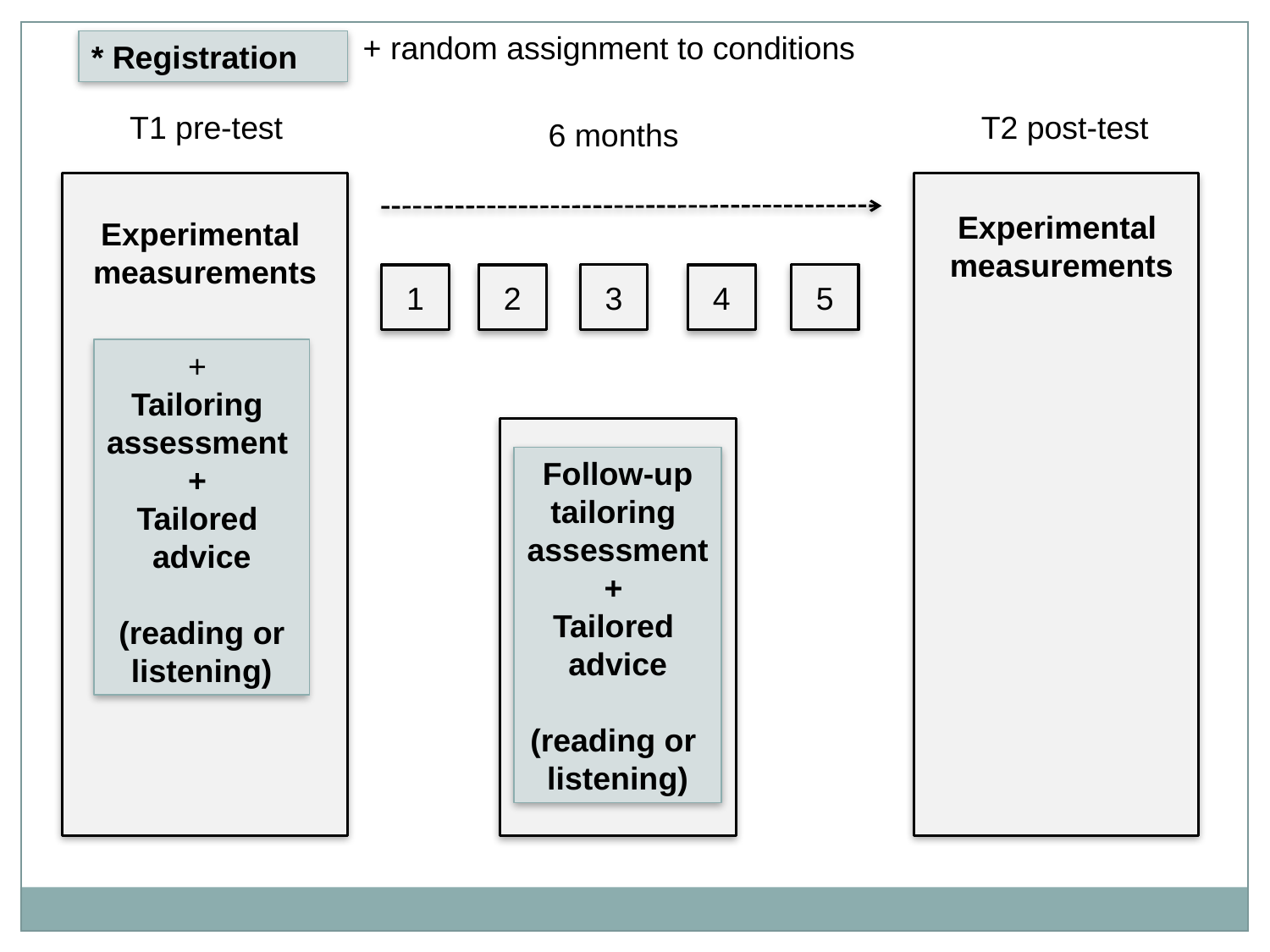

+ random assignment to conditions
* Registration
T1 pre-test
T2 post-test
6 months
Experimental
measurements
Experimental
measurements
3
5
1
2
4
+
Tailoring
assessment
+
Tailored
advice
(reading or
listening)
Follow-up
tailoring
assessment
+
Tailored
advice
(reading or
listening)

## Slide 3
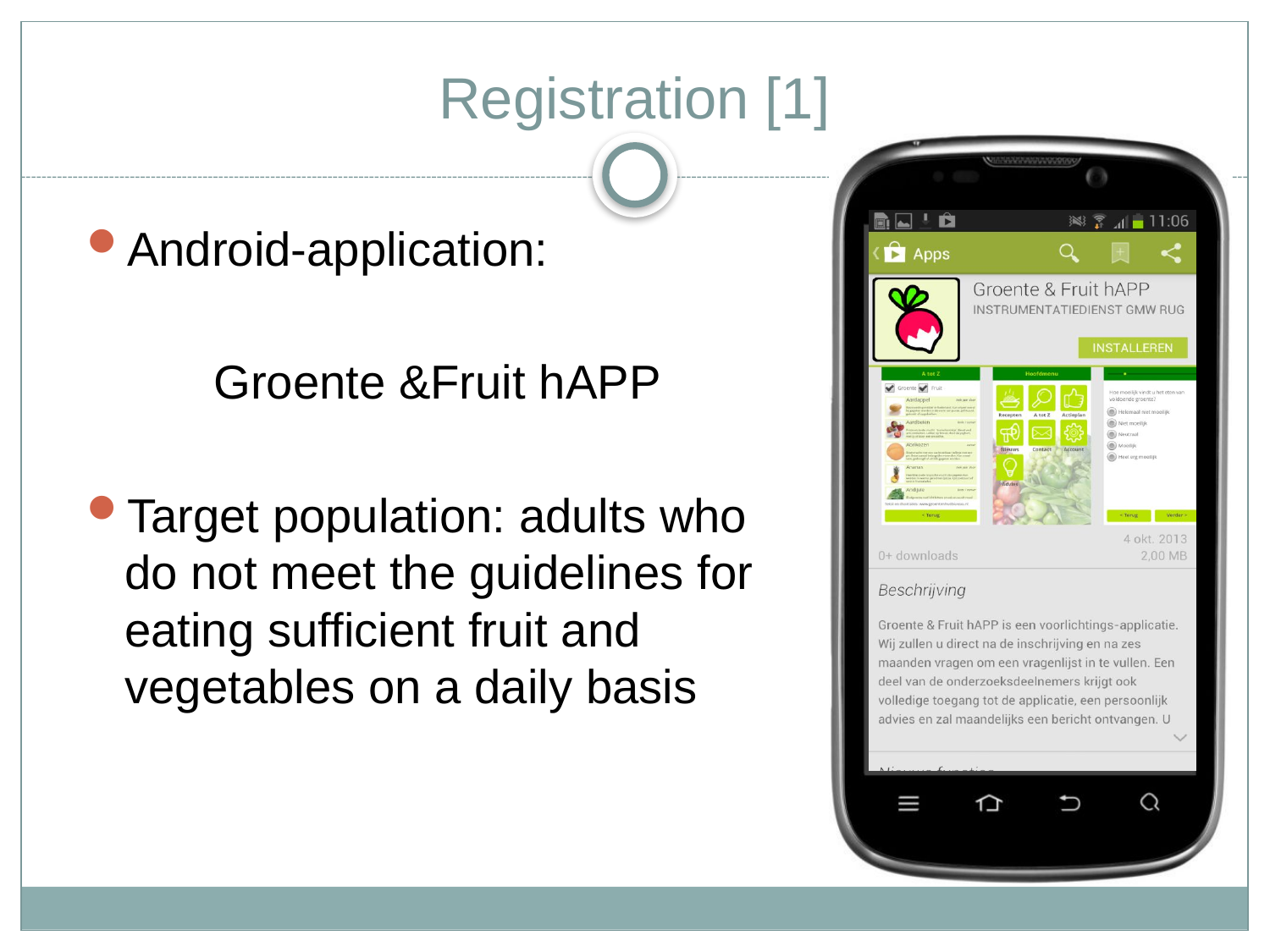

# Registration [1]
Android-application:
	Groente &Fruit hAPP
Target population: adults who do not meet the guidelines for eating sufficient fruit and vegetables on a daily basis

## Slide 4
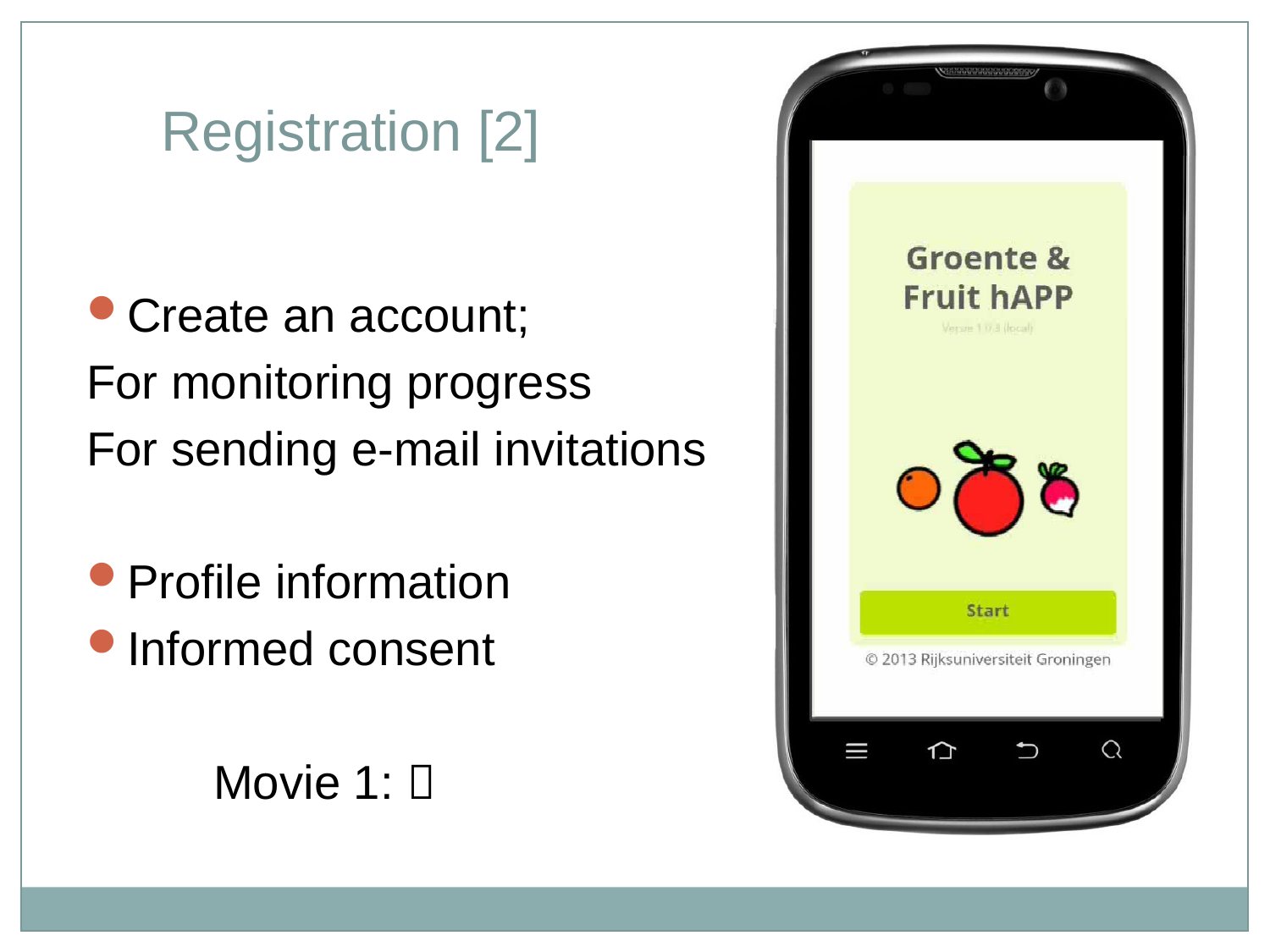

Registration [2]
Create an account;
For monitoring progress
For sending e-mail invitations
Profile information
Informed consent
	Movie 1: 

## Slide 5
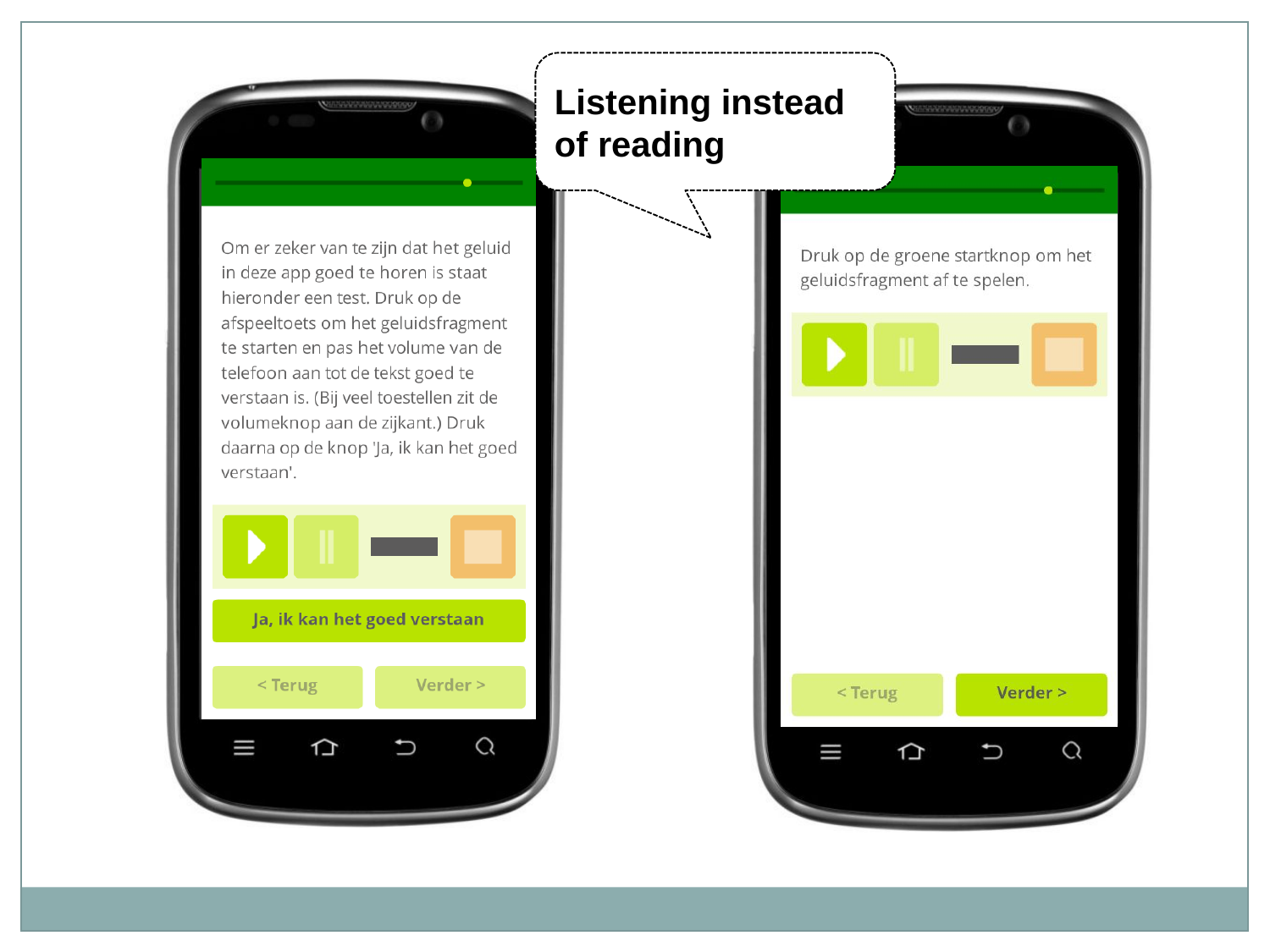

Listening instead of reading

## Slide 6
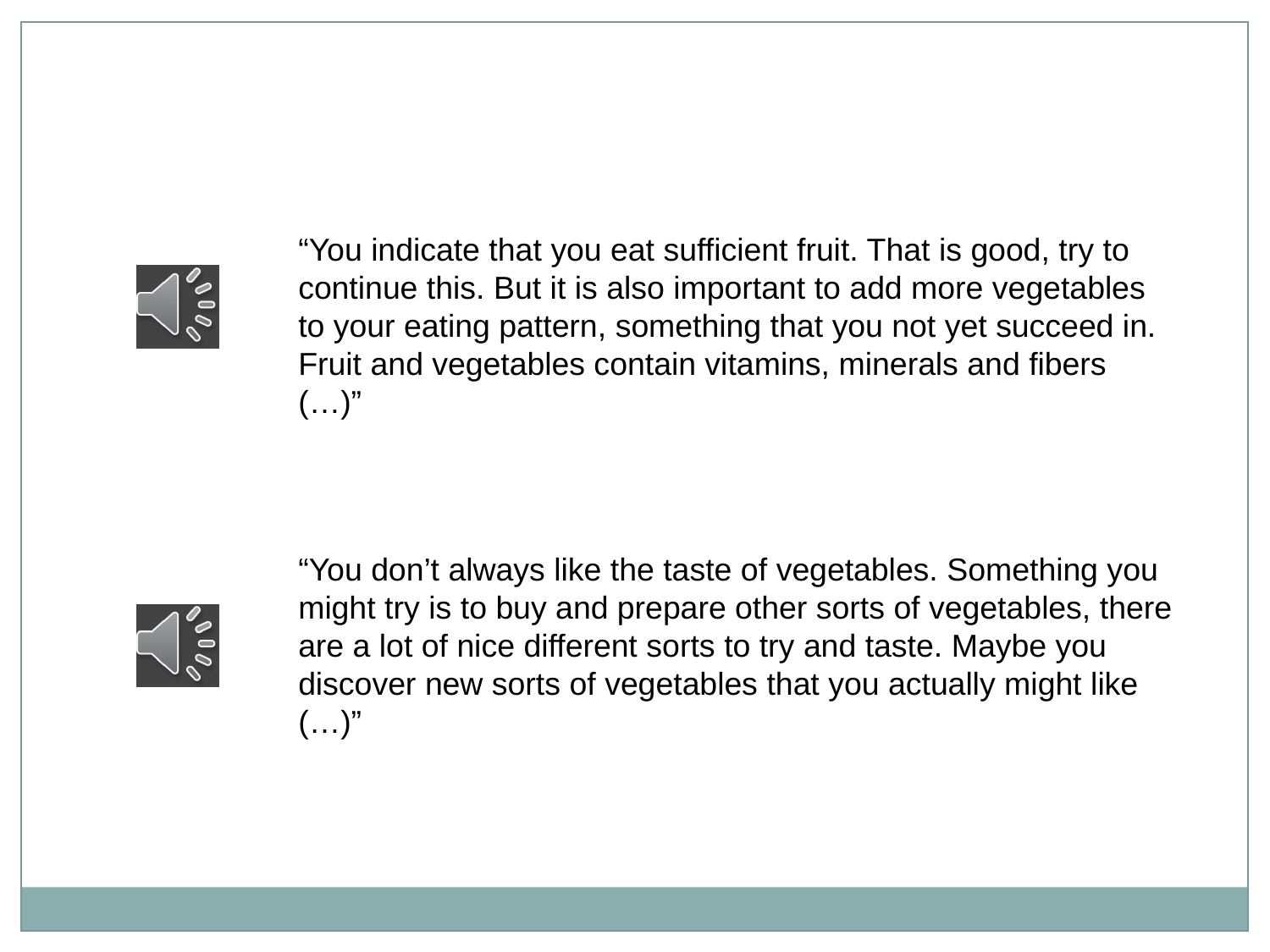

“You indicate that you eat sufficient fruit. That is good, try to continue this. But it is also important to add more vegetables to your eating pattern, something that you not yet succeed in. Fruit and vegetables contain vitamins, minerals and fibers (…)”
“You don’t always like the taste of vegetables. Something you might try is to buy and prepare other sorts of vegetables, there are a lot of nice different sorts to try and taste. Maybe you discover new sorts of vegetables that you actually might like (…)”

## Slide 7
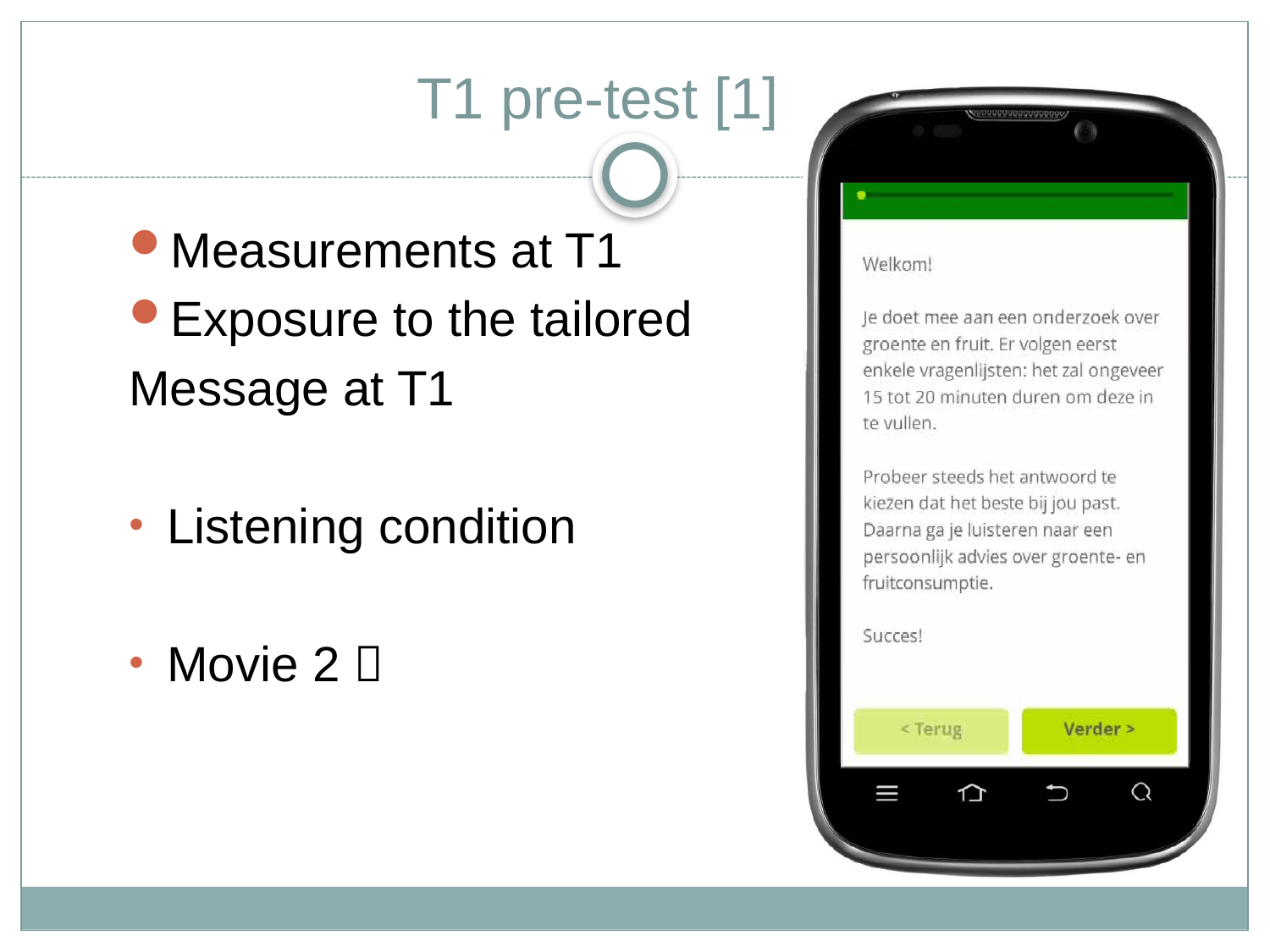

# T1 pre-test [1]
Measurements at T1
Exposure to the tailored
Message at T1
Listening condition
Movie 2 

## Slide 8
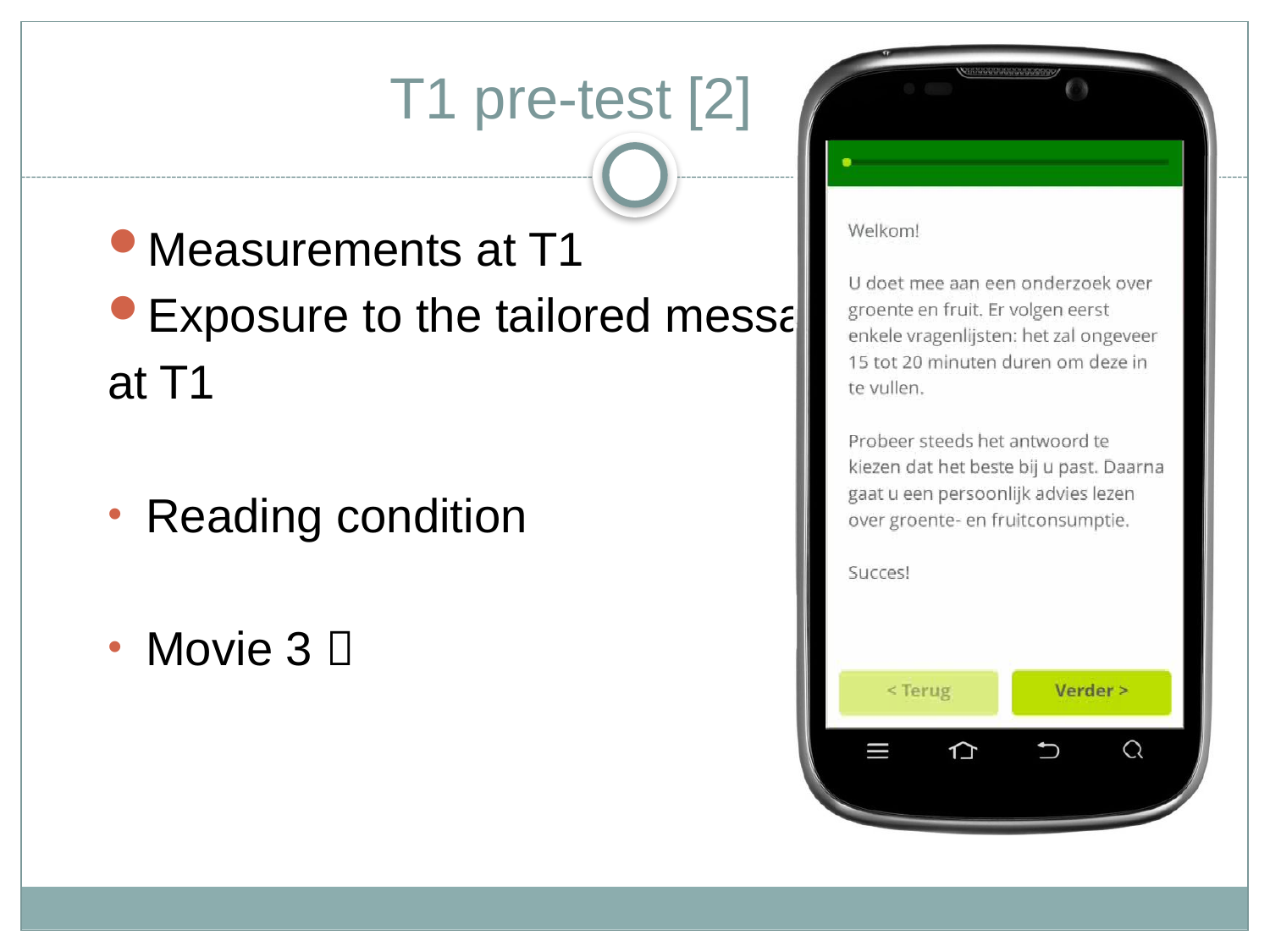

# T1 pre-test [2]
Measurements at T1
Exposure to the tailored message
at T1
Reading condition
Movie 3 

## Slide 9
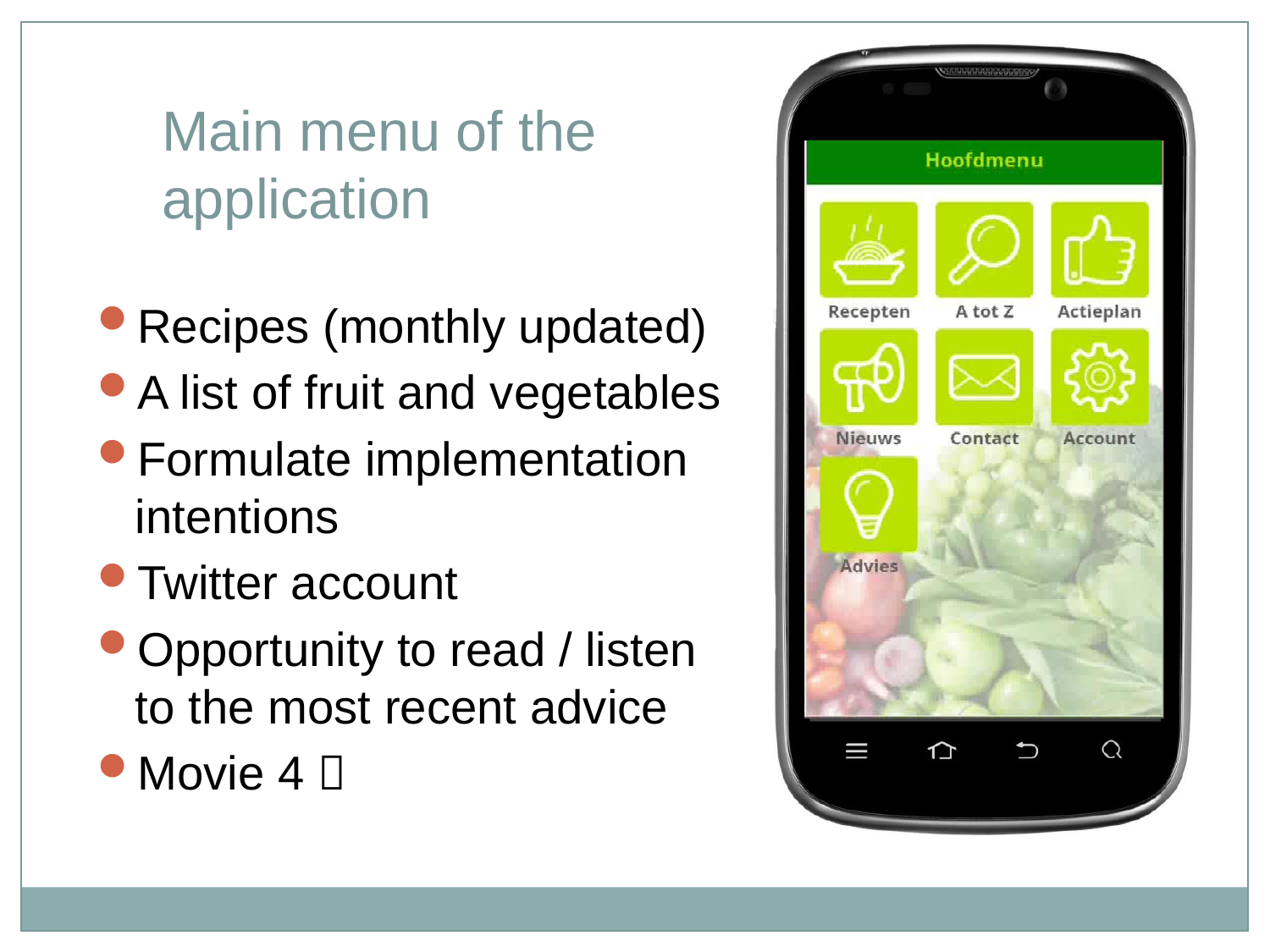

Main menu of the
application
Recipes (monthly updated)
A list of fruit and vegetables
Formulate implementation intentions
Twitter account
Opportunity to read / listen to the most recent advice
Movie 4 

## Slide 10
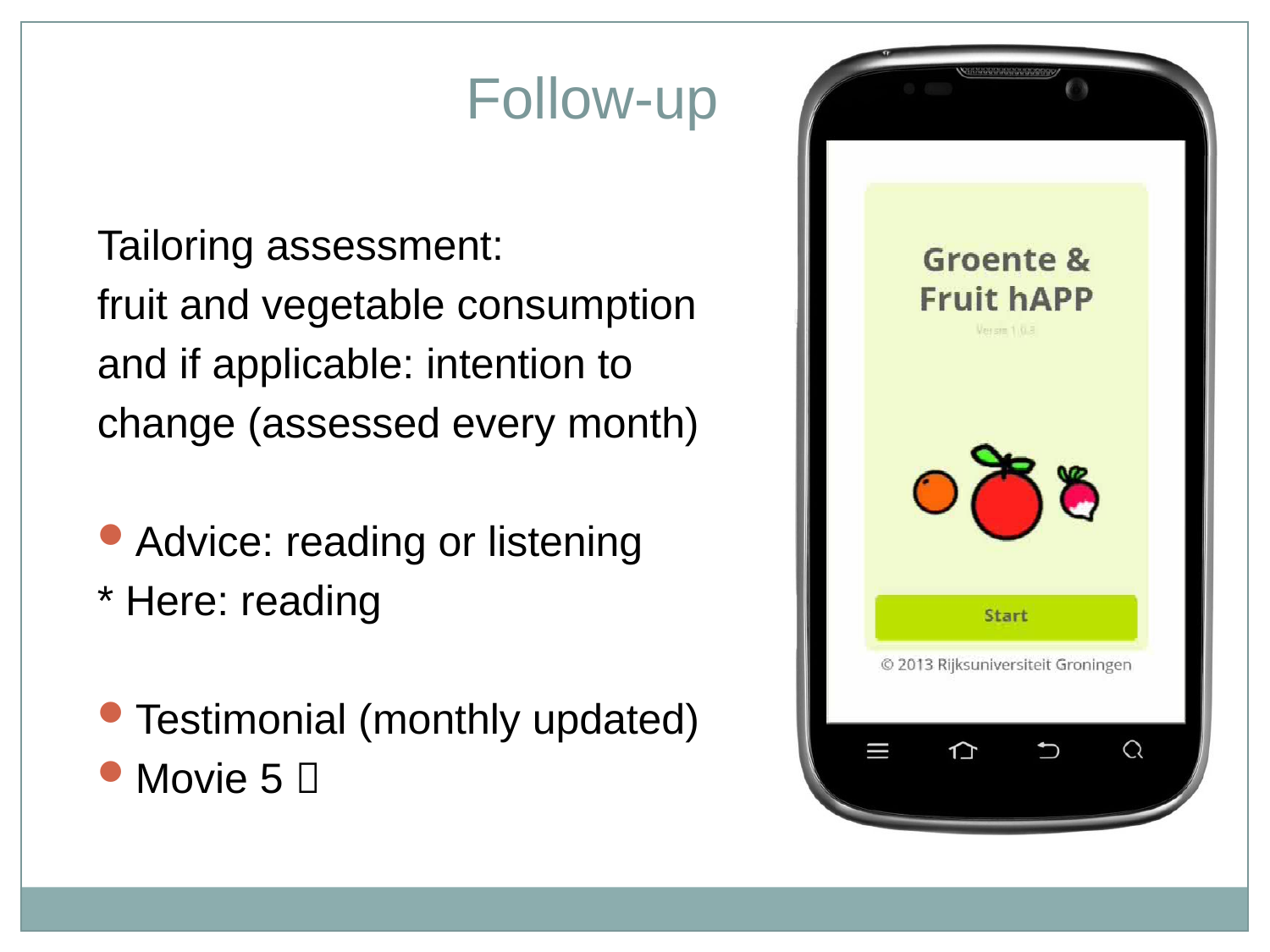

Follow-up
Tailoring assessment:
fruit and vegetable consumption
and if applicable: intention to
change (assessed every month)
Advice: reading or listening
* Here: reading
Testimonial (monthly updated)
Movie 5 

## Slide 11
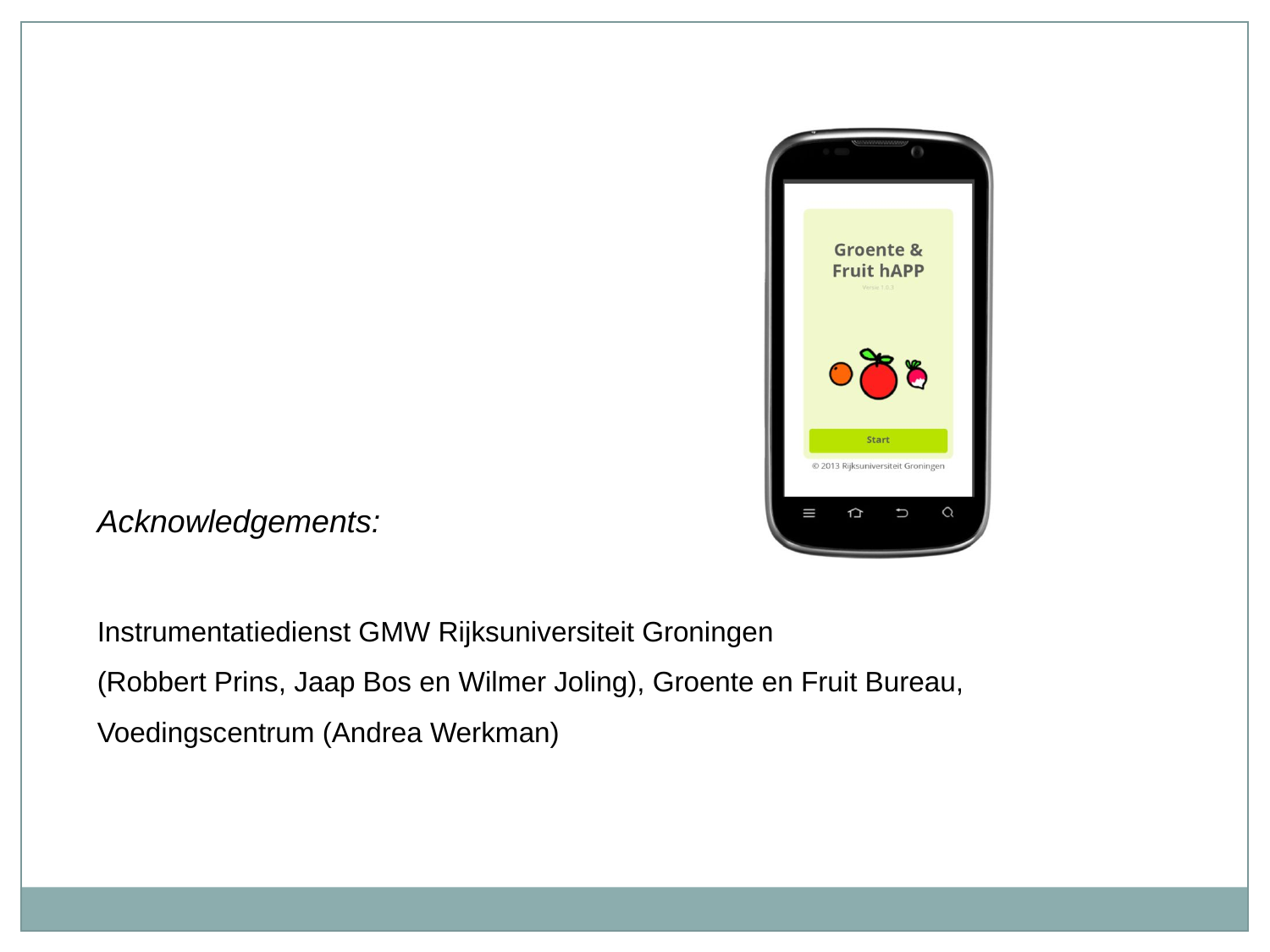

Acknowledgements:
Instrumentatiedienst GMW Rijksuniversiteit Groningen
(Robbert Prins, Jaap Bos en Wilmer Joling), Groente en Fruit Bureau, Voedingscentrum (Andrea Werkman)
